# Analysis of article screening and data extraction performance by an AI systematic literature review platform

**DOI:** 10.3389/frai.2025.1662202

**Published:** 2025-11-20

**Authors:** Kelsie Cassell, Abiodun Ologunowa, Majid Rastegar-Mojarad, Bianca Chun, Yi-Ling Huang, Dong Wang, Nicole Cossrow

**Affiliations:** 1 Merck & Co., Inc., Rahway, NJ, United States; 2 College of Pharmacy, University of Rhode Island, Kingston, RI, United States; 3 IMO Health, Rosemont, IL, United States

**Keywords:** artificial intelligence, systematic literature review, data extraction, reviewer workload, health technology assessment, large language models

## Abstract

**Background:**

Systematic literature reviews (SLRs) are critical to health research and decision-making but are often time- and labor-intensive. Artificial intelligence (AI) tools like large language models (LLMs) provide a promising way to automate these processes.

**Methods:**

We conducted a systematic literature review on the cost-effectiveness of adult pneumococcal vaccination and prospectively assessed the performance of our AI-assisted review platform, Intelligent Systematic Literature Review (ISLaR) 2.0, compared to expert researchers.

**Results:**

ISLaR demonstrated high accuracy (0.87 full-text screening; 0.86 data extraction), precision (0.88; 0.86), and sensitivity (0.91; 0.98) in article screening and data extraction tasks, but lower specificity (0.79; 0.42), especially when extracting data from tables. The platform reduced abstract and full-text screening time by over 90% compared to human reviewers.

**Conclusion:**

The platform has strong potential to reduce reviewer workload but requires further development.

## 1 Introduction

Systematic literature reviews (SLRs) are recognized as the most rigorous form of evidence review and synthesis (Aromataris and Pearson, 2014; Clarke and Chalmers, 2018; Grant and Booth, 2009; Munn et al., 2018). In the health economics field, SLRs on the relative cost-effectiveness of different interventions are increasingly used in health care system decision-making (Anderson, 2010; Jacobsen et al., 2020; Luhnen et al., 2018; Mandrik et al., 2021). However, SLRs require substantial investment of time and resources (Allen and Olkin, 1999; Borah et al., 2017; Bullers et al., 2018; Michelson and Reuter, 2019; Shemilt et al., 2016). Best-practice guidelines strongly recommend that SLR tasks be independently executed by at least 2 expert reviewers, which reduces the error rate and improves quality but also increases the overall workload (Higgins et al., 2024; Gartlehner et al., 2020; Page et al., 2021; Shemilt et al., 2016). These time- and labor-intensive processes can also introduce reviewer errors (Clark et al., 2021; Wang et al., 2020), as well as long gaps between SLR initiation and final publication (Beller et al., 2013; Borah et al., 2017). Long production times can in turn affect the longevity of the findings: an estimated 7% of SLRs are already out of date (defined as availability of new findings that would affect the conclusions of the synthesis) at the time of publication (Shojania et al., 2007). “Living SLRs,” a dynamic review format that allows ongoing online updates, have been proposed as a solution to this problem (Elliott et al., 2014; Wijkstra et al., 2021), but still require labor-intensive ongoing screening of newly published articles.

Many of the tasks involved in SLRs are amenable to automation using artificial intelligence (AI) (Beller et al., 2018; Bolaños et al., 2024; Michelson and Reuter, 2019; Shemilt et al., 2016; Tsertsvadze et al., 2015). Until recently, most work in this field used natural language processing (NLP) and similar text mining approaches (Blaizot et al., 2022; Bolaños et al., 2024; Cowie et al., 2022; de la Torre-López et al., 2023; van Dinter et al., 2021). Within the last 2 years, however, there has been extensive interest in the use of generative large language models (LLMs), which are more versatile and accessible than previous generations of AI tools (Lu et al., 2024; O’Connor et al., 2024; Sallam, 2023). Applications of LLMs in SLRs include construction of literature search terms (Alshami et al., 2023; Wang Z. et al., 2025; Wang et al., 2023), article screening (Akinseloyin et al., 2024; Alshami et al., 2023; Guo et al., 2024; Khraisha et al., 2024; Kohandel Gargari et al., 2024; Landschaft et al., 2024; Li et al., 2024; Matsui et al., 2024; Syriani et al., 2023; Tran et al., 2024; Wang Z. et al., 2025; Wang S. et al., 2024), data extraction (Alshami et al., 2023; Dunn et al., 2022; Gartlehner et al., 2024; Ghosh et al., 2024; Khraisha et al., 2024; Landschaft et al., 2024; Wang Z. et al., 2025), and article content synthesis/analysis (Alshami et al., 2023; Wang Z. et al., 2025).

Effective use of LLMs requires careful construction and iteration of the text “prompts” used to instruct the model. We recently developed a user-friendly LLM-based SLR platform, Intelligent Systematic Literature Review (ISLaR) 2.0, for semi-autonomous “human-in-the-loop” abstract and full-text article screening and data extraction (Wang et al., 2025a). Briefly, ISLaR 2.0 is based on ChatGPT4-Turbo (OpenAI, 2024) and incorporates an interface designed to help researchers who are experts in the topic of the SLR develop an effective LLM prompt by entering information such as the purpose of the SLR, article inclusion/exclusion criteria, and examples of relevant text and data elements (Wang et al., 2025a; Wang et al., 2025b). The platform automates the entire SLR process, from retrieving eligible articles in PubMed and Embrace to screening abstracts and full texts and extracting data from included studies. A key feature of ISLaR 2.0 is its human-in-the-loop design, which allows researchers to review screening results and iteratively refine their criteria as needed. Unlike many existing LLM-based tools that focus on a single task (e.g., search or extraction), ISLaR 2.0 integrates the full SLR workflow into one platform. We have performed initial tests of ISLaR’s performance by comparing its article selection and data extraction outputs to those of expert human reviewers in simulated SLR tasks (Wang et al., 2025a; Wang et al., 2025b). In these initial studies, our platform performed comparably to other published automated tools in terms of accuracy (abstract screening, 73.8–86.0%; full-text article screening, 78.3–85.7%; extraction of data from abstracts, 74.8–96.3%), and better than most other tools in terms of consistently high sensitivity (90.1–95.7%, 75.0–91.7%, and 90.3–97.6%, respectively) (Wang et al., 2025b).

This study aimed to prospectively assess the performance of ISLaR 2.0 compared to expert human reviewers in a full SLR, to identify areas where ISLaR can supplement human reviewers as well as those where human reviewers still outperform AI. In contrast to our previous work, which involved screening a subset of curated articles and extracting a limited number of data elements from study abstracts, this evaluation involved screening the full set of articles retrieved by the literature search and comprehensive extraction of relevant data from the full texts of included studies. The SLR’s research question focused on the cost-effectiveness of pneumococcal vaccination to prevent pneumococcal disease (PD) among adult’s ≥18 years of age, and specifically on studies that provide direct comparisons of vaccination costs and benefits. A cost-effectiveness analysis was chosen for this case study as SLRs of this kind involve unique challenges, including heterogeneity in study design, interventions, populations, and settings (Anderson, 2010; Husereau et al., 2022; Jacobsen et al., 2020; Mandrik et al., 2021). Our prospective case-study SLR thus required extraction of complex comparative outcomes in the correct context and permitted comprehensive comparison of AI and human reviewer decisions, providing a rigorous test of our platform’s performance and an important contribution to the literature on the automation of SLRs in the field of health economics.

## 2 Materials and methods

### 2.1 Search strategy, study selection criteria, and data extraction fields

The SLR was conducted in accordance with the Preferred Reporting Items for Systematic reviews and Meta-Analyses (PRISMA) 2020 and was pre-registered with the International Prospective Register of Systematic Reviews (PROSPERO; registration number CRD42024562351) (Cassell et al., 2024).

Search strings were manually developed for the PubMed and EMBASE literature databases to identify studies potentially related to the cost-effectiveness of pneumococcal vaccination among adult’s ≥18 years of age (Supplementary Table 1). Study selection was then based on the Population, Intervention, Comparison, Outcome, Time, and Study design (PICOTS) criteria listed in Table 1. We included peer-reviewed studies published in English between January 1, 2011 and January 1, 2023 that conducted cost-effectiveness analyses for any pneumococcal vaccine, compared to no vaccination or any standard-of-care vaccination, among adult’s ≥18 years of age. We excluded studies that reported only clinical outcomes with no cost component, or that reported cost–benefit, benefit ratio, or net benefit measures as the only health economic outcomes. Randomized controlled trials were also excluded, as were reviews, posters, published conference abstracts, preprints, and other non-primary and/or non-peer-reviewed publication types. Different criteria were applied at the abstract and full-text screening stages to emphasize sensitivity at the abstract screening stage and then apply more stringent exclusion criteria to the full texts. This approach is consistent with guidelines created by the Professional Society for Health Economics and Outcomes Research (ISPOR) for the conduct of SLRs that focus on cost-effectiveness outcomes (Mandrik et al., 2021; Wang et al., 2025a; Wang et al., 2025b). For example, the age-based population exclusion criterion applied at the abstract stage was designed to discard studies that included *only* populations <18 years of age; at the full-text screening stage, we discarded studies that included *any* population <18 years of age.

**Table 1:** Study inclusion and exclusion criteria.

Variable	Inclusion criteria	Screening stage	Exclusion criteria	Screening stage
Population	Human population eligible for pneumococcal vaccine (vaccine to prevent infection due to *Streptococcus pneumoniae*)	Abstract and full text	Studies on non-human subjects (such as mice)	Abstract and full text
	Study o*nly* includes adults or elderly 18 years and older	Full text	Study *only* includes infants, children or adolescent populations, ages, 0–17 years	Abstract
			Study includes infants, children, and adolescents, 0–17 years	Full text
Intervention	Any vaccine to prevent *streptococcus pneumoniae* (such as PCV, PPSV, pneumococcal conjugate vaccine, or pneumococcal polysaccharide vaccine)	Abstract and full text	NA	NA
Comparison	Any vaccine comparison (for example, vaccine to unvaccinated, PCV vaccinated to PPSV23 vaccinated)	Full text	NA	NA
Outcome	Studies with full healthcare cost-effectiveness analysis (CEA) which can include outcomes such as: Incremental cost-effectiveness ratios (ICER) Medical costs (direct or indirect) Indirect costs (societal, resource use, productivity) Quality-adjusted life year (QALY), Life-year (LY) Cases and deaths averted by intervention	Full text	If the only outcomes presented are cost–benefit, benefit ratio or net benefit measures	Full text
			Studies that only report clinical efficacy, safety, vaccine efficacy, vaccine effectiveness	Full text
Time	Publication date between January 1, 2011 and January 1, 2023	Abstract and full text	NA	NA
Study design	Cost-effectiveness analyses (for example, Markov model analyses)	Full text	Randomized control trials and clinical trials	Abstract and full text
	Quality-of-life studies using generic and disease-specific measures *with* cost components	Full text	Reviews (such as systematic literature reviews, targeted literature reviews, narrative literature reviews, and meta-analysis)	Abstract and full text
			Vaccine effectiveness, vaccine impact, vaccine efficacy, and case–control studies	Full text
			Budget-impact OR cost–benefit analyses (for example, studies that assign a dollar value to health outcomes)	Full text
			Articles reporting cost estimates that are not based on data (e.g., commentaries referencing cost burden)	Full text
			Posters, preprints, consensus reports, editorials, commentaries, case studies, news articles	Full text
Other	English language	Abstract and full text	NA	NA

NA, not applicable (no exclusion criteria specified for this variable).

The selection of relevant data elements for extraction was guided by the Consolidated Health Economic Evaluation Reporting Standards (CHEERS) 2022 statement (Husereau et al., 2022). The data extracted from full-text articles included study design parameters and outcomes, categorized into 23 text-based fields (i.e., data expected to be found in the text of the publication, such as model type and study limitations) and 18 table-based fields [i.e., data expected to be found in the study’s tables, such as quality-adjusted life years (QALYs) and incremental cost-effectiveness ratios (ICERs); Supplementary Table 2]. Extracted data were classified as follows:

- Element: data field of interest, such as “model type”, “number of deaths”, or “conclusion”.
- Value: exact value of each element (e.g., “1 year” for the “model cycle” element).
- Study cohort: study subpopulation specific to a given element and its value (e.g., individuals receiving study vaccine versus individuals receiving comparator vaccine, or adults <65 years of age versus adults ≥65 years of age; left blank for elements and values that applied to the entire study, such as model type).

For text-based values, we also extracted the corresponding text spans from full-text articles; i.e., direct quotations of the section of text within the full-text publication from which each element and/or its value was identified. This quoted text span was used to identify potential explanations for errors made by ISLaR. Text spans were not available for data extracted by ISLaR from study tables; any discrepancies in table-based data elements were therefore resolved manually by the human reviewer team.

No assessment of quality or bias, meta-analysis, or other detailed content analysis of the included studies was performed.

### 2.2 ISLaR prompt development

ISLaR 2.0 is an LLM-based tool designed by IMO Health, Rosemont, IL, US, in collaboration with the study’s authors, to conduct SLRs (Wang et al., 2025a). The tool is based on ChatGPT4-Turbo (GPT-4-turbo-2024-04-09) (OpenAI, 2024).

The text prompt included the full set of instructions for conducting the SLR, and a zero-shot strategy was employed (Supplementary Table 3). The prompt was constructed based on information on the SLR’s study PICOTS (Population, Intervention, Comparator, Outcome, Time, Study design) inclusion and exclusion criteria and data extraction framework, provided by authors who are experts in PD and pneumococcal vaccination via ISLaR’s semi-structured user interface (Table 1). The prompt also included background information such as definitions of pneumococcal vaccination, relevant study types, PD outcomes, vaccines, and other study variables. In addition, the subject matter expert authors provided examples of study text spans for each data field of interest, which were included to improve ISLaR’s ability to recognize and extract relevant data (Supplementary Table 2). The prompt based on this information was developed iteratively (2 versions) using a human-in-the-loop approach. After initial assessment of the screening decisions made by ISLaR using the first version of the prompt, 2 modifications were made. The first version of the population inclusion/exclusion criterion prompt stated “Exclude studies if they include infants, children, or adolescent populations, ages 0–17 years”; however, ISLaR failed to accurately apply this criterion, and so the prompt was updated in the second version to “Make sure to exclude the article if it involves any infants or children or adolescents or any participants below 18 years of age”. To avoid the conflations of non-Anglophone study locations with non-English-language publications that we observed with the first version of the prompt, the English language criterion was also updated, from “English language only” to “Studies in English language only”; in addition, the country acronyms that were originally included in the background knowledge prompt were removed, as they may have contributed to some of these errors. All study results reported below reflect the use of the second and final iteration of the prompt.

### 2.3 Study selection

The human reviewer team searched PubMed and EMBASE manually and entered the titles and abstracts of all studies into Microsoft Excel. A single reviewer identified and removed duplicates based on PubMed ID (PMID), or title and first author for studies not indexed in PubMed. Three reviewers then independently screened the abstracts of the remaining unique studies, applying the subset of PICOTS criteria that were assessed during the abstract screening stage and recording all reasons for exclusion. Any discrepancies between reviewers were resolved following independent review by an additional researcher. The full texts of the studies that passed the abstract screening (excluding any supplementary materials, which could not be assessed by ISLaR) were then independently screened by two reviewers using the subset of PICOTS criteria that were assessed at this stage; again, reasons for study exclusion were recorded. Any discrepancies between these reviewers were resolved following secondary review by two independent researchers. All study team members manually recorded the time taken to complete each screening task.

ISLaR searched PubMed using public application programming interfaces (APIs)—specifically, “E-utilities” for the abstract search and PubMed Central APIs for full text retrieval (National Library of Medicine National Center for Biotechnology Information, n.d.)—and EMBASE using the same standard search interface that was used by the human reviewers. The results were reduplicated based on PMID when available, or digital object identifier (DOI) for articles without a PMID. At the abstract screening stage, ISLaR assessed the study’s title and abstract. At the full-text screening and data extraction stages, ISLaR assessed the entire text of the published study, excluding any supplementary material. For studies where the full text was not available on the journal’s website, Amazon Textract was used to convert manually downloaded study PDF files to text (Amazon Web Services, Inc, n.d.). At both screening stages, ISLaR categorized each study as relevant (included) or irrelevant (excluded) and recorded the reason(s) for exclusion of each study. For this study, ISLaR was run fully autonomously, without any human-in-the-loop intervention.

### 2.4 Data extraction

Four human reviewers independently extracted relevant data from each remaining study into a standard Excel template containing the data fields listed in Supplementary Table 2. Any discrepancies were resolved following secondary review by an additional researcher. For each data element of each included study (e.g., number of deaths due to a given PD), the reviewers extracted the applicable study cohort (e.g., subgroup of the overall study cohort stratified by age or other characteristic, such as “adults 18–64 years of age”) and value (e.g., “1,516 deaths”), and, if the data were extracted from text rather than a table, the relevant text span from the study (e.g., “1,516 deaths due to PD were recorded among adults 18–64 years of age”).

ISLaR also extracted all available data elements and their corresponding value and study cohort, as well as the applicable text span from the study. Missing values were left blank by both the human reviewer team and ISLaR. All extracted data and metadata (e.g., reasons for study exclusion) were exported from ISLaR’s user interface into Excel for analysis. As with screening, data extraction was performed entirely by ISLaR. A third human reviewer then compared the values extracted by ISLaR and by the human reviewers with the values reported in the full texts and made the final decision on whether each value was correct.

### 2.5 Comparative analysis

Study selection performance was assessed separately for the abstract screening and full-text screening stages of the process, with the human reviewers’ selections considered the gold standard. Human reviewers included subject matter experts in the field of pneumococcal vaccine epidemiology and economic evaluation. For analysis of screening results, each study was categorized as a true positive (TP; study included by human reviewers and ISLaR), true negative (TN; study excluded by human reviewers and ISLaR), false positive (FP; study excluded by human reviewers but included by ISLaR), or false negative (FN; study included by human reviewers but excluded by ISLaR). For the assessment of ISLaR’s data extraction performance, we randomly selected 21 studies (33% of the original TN set of studies, before manual refinement) that were classified as TPs at the full-text screening stage. The data extracted by ISLaR from these 21 studies were compared to the values present in the full texts of the published studies, which were considered the gold standard. Each ISLaR-extracted value was assessed as a TP (ISLaR extracted the correct value from the text), TN (ISLaR output “not applicable” (“NA”) or blank when a value was not present in the text), FP (ISLaR provided an incorrect value, or provided any value when a value was not present in the text), or FN (ISLaR output “NA” or left the field blank for a value that was present in the text).

For each study selection and data extraction performance comparison, the TP/TN/FP/FN categories were used to assess ISLaR’s performance via the following metrics:

- Accuracy: (TP+TN)/(TP+FP+TN+FN)
- Precision/positive predictive value (PPV): TP/(TP+FP)
- Recall/sensitivity: TP/(TP+FN)
- Specificity: TN/(TN+FP)
- F1 value: (2×precision×recall)/(precision+recall)
- F2 value: (5×precision×recall)/(4×precision+recall)
- Matthew’s correlation coefficient (MCC): $\frac{\mathrm{TP}\times \mathrm{TN}-\mathrm{FP}\times \mathrm{FN}\;}{\sqrt{\left(\mathrm{TP}+\mathrm{FP})\times (\mathrm{TP}+\mathrm{FN})\times (\mathrm{TN}+\mathrm{FP})\times (\mathrm{TN}+\mathrm{FN}\right)}}$
- Work saved over sampling at 95% recall (WSS@95%): $\frac{\mathrm{TN}+\mathrm{FN}}{N}-0.05$

The nature of each discrepancy was also noted (for example, which of the PICOTS criteria was/were misclassified for each FN or FP study).

ISLaR-extracted data were also compared to the corresponding human-extracted data to identify examples of both ISLaR-specific and human-specific errors compared to the gold-standard full texts, but no performance metrics were calculated for this comparison. Inter-rater agreement of abstract and full text screening was assessed through Cohen’s Kappa (Supplementary Table 4).

## 3 Results

### 3.1 Study selection

**Figure 1:**
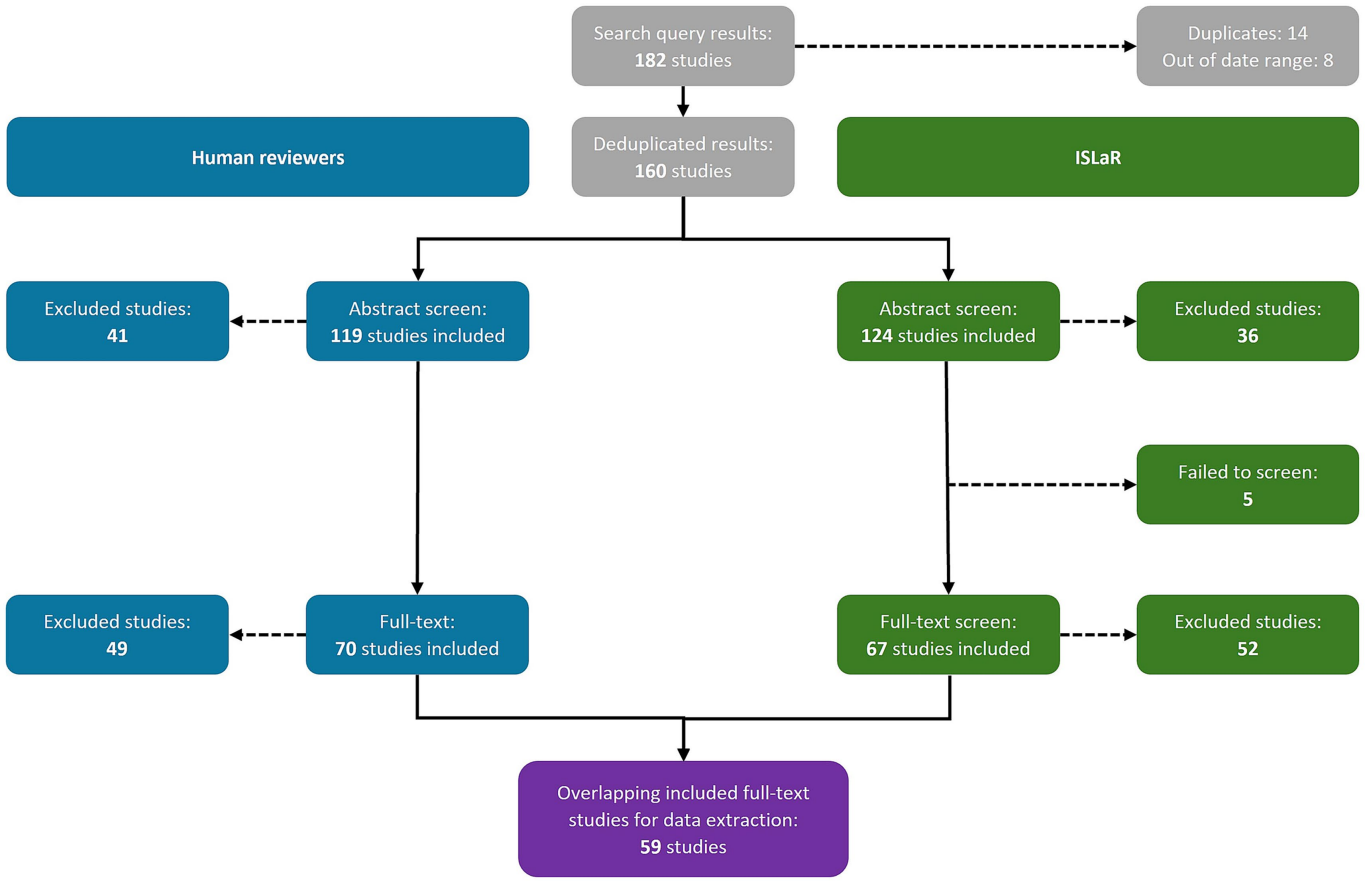
Study selection results. ISLaR, intelligent systematic literature review.

The database search returned 182 studies: 145 from PubMed and 37 from EMBASE (Supplementary Table 1). Both the human reviewers and ISLaR correctly identified and removed 14 duplicates and 8 studies published outside the desired date range, retaining a total of 160 studies for abstract screening (Figure 1). All studies, and their classifications at the abstract and full-text screening stages, are listed in Supplementary Table 5.

#### 3.1.1 Abstract screening

The human reviewers included 119 study abstracts and excluded 41, while ISLaR included 124 study abstracts [107 true positives (TPs) and 17 false positives (FPs), compared to the human reviewers] and excluded 36 [24 true negatives (TNs) and 12 false negatives (FNs); Figure 1; Table 2]. ISLaR thus had high recall/sensitivity (0.90), accuracy (0.82), precision/positive predictive value (PPV; 0.86), and F1 value (0.88) for abstract screening, but lower specificity (0.59; Table 2). There were 22 discrepancies between the decisions of different human reviewers that had to be resolved via independent review by an additional researcher; in these cases, the independent reviewer’s decision was considered correct moving forward. The studies for which inter-reviewer discrepancies occurred included 4/17 FP and 2/12 FN abstracts [i.e., 6/29 (20.7%) of all abstract screening errors made by ISLaR].

**Table 2:** ISLaR performance metrics.

Stage		Records				Performance metrics							
		TP	TN	FP	FN	Accuracy	Precision/PPV	Specificity	Recall/sensitivity	F1	F2	MCC	WSS @95% recall
Abstract screening		107	24	17	12	0.82	0.86	0.59	0.90	0.88	0.89	0.51	0.18
Full-text screening		59	31	8	6	0.87	0.88	0.79	0.91	0.89	0.90	0.71	0.31
Full-text data extraction^a^	Study text	236	27	37	6	0.86	0.86	0.42	0.98	0.92	0.95	0.52	0.06
	Tables	201	44	132	117	0.50	0.60	0.25	0.63	0.62	0.62	−0.12	0.28

ISLaR, intelligent systematic literature review; FN, false negative; FP, false positive; PPV, positive predictive value; TN, true negative; TP, true positive; MCC, Matthew’s correlation coefficient; WSS@95%, work saved over sampling at 95% recall. ISLaR’s performance was compared to that of the human reviewer team unless otherwise stated.

a Compared to the full texts of TN studies after full-text screening.

ICER, incremental cost-effectiveness ratio; LY, life year; QALY, quality-adjusted life year; RCT, randomized controlled trial. More than one incorrect reason for exclusion was given for some studies.

In the review conducted by ISLaR, the primary reasons for the false exclusion of studies are outlined in Table 3. At the abstract screening stage, five studies were erroneously identified as randomized controlled trials (RCTs), either due to ISLaR inferring an incorrect definition of an RCT, or confusion between the design of the study in question and a mention of a separate RCT publication within the background section of the abstract. Additionally, we observed two cases where English-language study abstracts were incorrectly identified as non-English language (due to non-Anglophone study location), despite re-wording the language criterion used in the final version of the prompt to attempt to avoid this issue. Among 12 FN abstracts, eight had a secondary exclusionary reason that was either clearly wrong or a misinterpretation of the Population, Intervention, Comparison, Outcome, Time, and Study design (PICOTS) criteria. Examples of secondary reasons for exclusion provided for FN abstracts included classification of case–control studies and cost-effectiveness analyses as RCTs, classification of primary data analyses as reviews, failure to recognize that adult’s ≥60 years of age were a population eligible for pneumococcal vaccination, and failure to recognize that a study was published in the English language. Incorrect secondary exclusion criteria were also identified for 10/24 TN abstracts.

**Table 3:** Qualitative study exclusion errors made by ISLaR.

Reason given for exclusion	Number of studies	Example rationale(s) provided by ISLaR	Most likely explanation for error
Abstract screening stage			
Is a clinical trial	5	“The study is a randomized controlled trial, which is listed in the exclusion criteria. Therefore, it is not eligible” Smith et al. (2017)	An RCT was mentioned in the first sentence of the abstract of a non-RCT study
		“The study also appears to be a cost-effectiveness analysis, which could be considered a type of clinical trial, another exclusion criterion” Pugh et al. (2020)	Incorrect definition of a clinical trial was used
Not in English language, or language not specified	2	“Additionally, the study was conducted in Brazil, not in an English-speaking country, which is another exclusion criterion” de Soarez et al. (2015)	Confusion between Anglophone study location and English language study
Human population eligible for vaccination	2	“Additionally, the study does not specify that it is conducted on a human population eligible for pneumococcal vaccine” Wateska et al. (2018)	Study population was eligible for pneumococcal vaccination based on comorbidities, but this eligibility was not explicitly stated in the study abstract
Study of children only	1	“The study focuses on pediatric population which is excluded as per the criteria” Eythorsson et al. (2021)	Study evaluated the effects of pediatric pneumococcal vaccination on all age groups, including adults, but only pediatric age groups were mentioned in the abstract
Full-text screening stage			
Does not include full health cost-effectiveness analysis outcomes	3	“The article is excluded because it does not meet the inclusion criteria for outcomes. The article focuses on value of information analysis and does not provide a full healthcare cost-effectiveness analysis including outcomes such as ICER, medical costs, indirect costs, QALY, LY, cases and deaths averted by intervention as specified in the inclusion criteria” Wateska et al. (2021)	The study did report QALY values, but not the full set of all possible outcomes for a cost-effectiveness analysis
		“The article is excluded because it focuses on budget-impact analysis, which is specifically mentioned in the exclusion criteria under “Other” as studies that are budget-impact or cost–benefit analyses are to be excluded” Liguori et al. (2014)	Two studies included by the human reviewers were excluded by ISLaR due to being a budget-impact analysis. Following data extraction by the human reviewers, it was decided that these studies should indeed have been excluded during full-text screening
Age group	2	“The Australian infant pneumococcal vaccination program is mentioned, which involves participants under 18 years of age, thus not meeting the population criteria specified in the exclusion criteria” Chen et al. (2018)	ISLaR appears to have confused the intervention (pediatric vaccination) with the study population (adults protected via herd immunity from pediatric vaccination)
Does not include relevant disease outcomes	1	“The article is excluded because it focuses on the prevention of acute coronary syndrome (ACS) using the pneumococcal polysaccharide vaccine (PPV), which is not directly related to the prevention or treatment of pneumococcal disease conditions listed in the inclusion criteria. The outcomes discussed in the article (cost-effectiveness in preventing ACS) do not align with the outcomes required by the inclusion criteria, which focus on pneumococcal disease-specific outcomes such as healthcare cost-effectiveness analysis related to pneumococcal disease” Ren et al. (2021)	The outcomes criteria input into ISLaR did not exclude any specific diagnoses, but the prompt defined a list of possible outcomes that did not include ACS. ISLaR likely conflated this list of possible outcomes with strict inclusion criteria

ICER, incremental cost-effectiveness ratio; LY, life year; QALY, quality-adjusted life year; RCT, randomized controlled trial. More than one incorrect reason for exclusion was given for some studies.

ISLaR’s FP abstract selections also included errors in recognizing English-language studies (potentially due to some non-English-language studies having an English-language version of the abstract), as well as posters, preprints, and non-primary studies such as editorials, commentaries, and news articles that were correctly excluded by the human reviewers. Human reviewers also excluded nine abstracts that did not assess cost-effectiveness outcomes in an adult population, but that ISLaR erroneously included; in six cases the human reviewers’ exclusion decision was based on information that was also available to ISLaR (for example, use of the words “in children” in the study title), whereas in the other cases the decision was based on expert knowledge that had not been included in the ISLaR prompt (for example, knowledge that the study’s intervention vaccine is only licensed for use in pediatric populations).

#### 3.1.2 Full-text screening

At the full-text review stage, the human reviewers excluded 49 additional studies, retaining 70 for data extraction and detailed review (Figure 1). In contrast, ISLaR was unable to assess the full text of five articles and excluded an additional 52 studies following full-text review. When comparing the subset of studies that were assessed by both the human reviewer team and ISLaR at the full-text stage, ISLaR excluded 37 studies (31 TNs and six FNs compared to the human reviewer gold standard) and retained 67 (59 TPs and 8 FPs) for data extraction (Table 2). ISLaR’s recall (0.91), accuracy (0.87), precision (0.88), and F1 value (0.89) at the full-text screening stage were thus slightly higher than the corresponding values from the abstract screening phase. As expected due to the use of more stringent inclusion and exclusion criteria for the full-text screen, there was a much greater improvement in the specificity score: from 0.59 at the abstract screening phase to 0.79 at the full-text screening phase. There were 16 discrepancies between the decisions of different human reviewers; the studies for which these discrepancies occurred included 3/8 FP and 2/6 FN full-text studies [i.e., 5/14 (35.7%) of the full-text screening errors made by ISLaR].

Two full-text studies were erroneously excluded by ISLaR due to inaccurate classification of the study population’s age group (for example, when an infant vaccination program was mentioned in a study that included an assessment of the impact of pediatric vaccination on adults via herd immunity) and one because of inaccurate classification of the study’s outcomes (Table 3). In addition, three FN studies were excluded due to lack of relevant cost-effectiveness analysis outcomes. After data extraction the human reviewer team concluded that two of these three studies were budget-impact analyses that should indeed have been excluded during full-text screening (i.e., these studies were actually TNs). The ISLaR accuracy and recall metrics for full-text screening listed in Table 2 are thus slight underestimates.

As an informal sensitivity analysis, we prompted ISLaR to assess the full texts of all studies it had excluded at the abstract screening phase. Only 3/36 (8.3%) of these excluded studies were considered relevant to the SLR when reassessed using the full text.

#### 3.1.3 Human–ISLaR comparison

Cohen’s kappa values comparing human–human and human–ISLaR screening results are shown in Supplementary Table 4. At the abstract screening stage, agreement between human reviewers was moderate (*κ* = 0.65) and higher than the agreement between ISLaR and the composite human decision (*κ* = 0.50). At the full-text screening stage, the human–human agreement (*κ* = 0.75) was similar to the human–ISLaR agreement (*κ* = 0.73). It should be noted that the human–ISLaR comparison was made against the composite human decision, in which discrepancies between human reviewers were resolved by a third reviewer.

### 3.2 Data extraction

A total of 59 TP full-text studies were included by both the human review team and ISLaR, and thus proceeded to the data extraction phase. The data extracted from 21 randomly selected studies from among this group were used to assess ISLaR’s performance. Compared to the full texts of the respective studies, ISLaR had high accuracy (0.86), precision/PPV (0.86), recall/sensitivity (0.98), and F1 value (0.92) when extracting data elements from study texts, but relatively low specificity (0.42; Table 2). Most FPs came from misattributing cost denomination years, where ISLaR pulled a year mentioned elsewhere in the text and incorrectly classified it as the monetary denomination year. For these text-based data extractions, ISLaR extracted the correct value 87.8% of the time when compared to the gold-standard values present in the studies’ full texts. However, it extracted the correct value 98.4% of the time compared to the values initially extracted by the human reviewers, due to errors and omissions in the human reviewers’ data extractions.

The model’s performance was worse across all metrics when extracting data from study tables rather than study texts (Table 2); overall, ISLaR extracted the correct data from Tables 48.3% of the time compared to the gold-standard values present in the studies’ full texts and 63.9% of the time compared to the human reviewers. Consistent with these results, ISLaR’s F2 and MCC values were high for text-based extractions (F2 = 0.95; MCC = 0.52), but much lower for table-based extractions (F2 = 0.62; MCC = −0.12). For table extractions, FPs generally reflected ISLaR extracting a value from the wrong cell of the relevant table. For example, ISLaR had difficulties accurately extracting study cohort information from large tables, especially when table subheadings were used to differentiate between results from different subpopulations, such as age groups. ISLaR also often recorded “NA” for a value that was present in a table. In addition, there were several instances of ISLaR extracting data from a cost-effectiveness Markov model input table, which in many studies includes similar variables and is formatted similarly to the outcomes data tables; this distinction was not included in ISLaR’s prompt. These extractions were only logged as errors in cases where ISLaR did not also provide an accurate data extraction from the study’s outcomes tables.

We identified several cases where ISLaR successfully extracted data elements that were not found or incorrectly extracted by one (but not both) human reviewer(s): ISLaR’s extracted value was correct for 72.9% of all single-reviewer errors in extracting text-based data, and 27.9% of all single-reviewer errors in extracting table-based data. For 10 data values, ISLaR correctly extracted a data element that was not identified (i.e., was marked as “NA”) by any human reviewer.

### 3.3 Time to completion

Abstract screening was completed in a mean of 2.99 s per abstract by ISLaR (at low user volume times, i.e., outside standard office hours) and 66.12 s per abstract by the human reviewers. At the full-text screening stage, the average time taken for ISLaR to screen each full text was 7.49 s, compared to 80.86 s for the human reviewers. The average reduction in review time was approximately 63 s for abstracts and 73 s for full-text review.

## 4 Discussion

In this study, we prospectively compared the performance of an LLM-based platform to that of expert human reviewers in an SLR on the cost-effectiveness of adult pneumococcal vaccination. ISLaR screened articles with high sensitivity, accuracy, and precision, but lower specificity, in 4.5 and 9.3% of the time taken by human reviewers to screen abstracts and full texts, respectively. In data extraction tasks, ISLaR performed markedly better across all metrics when extracting data elements from study texts compared to study tables. For 20.7% of ISLaR’s abstract screening errors, 35.7% of its full-text screening errors, and 61.0% of its data extraction errors, there was a corresponding discrepancy between human reviewers.

ISLaR’s time savings and performance metrics were broadly in line with those reported in other studies of LLM-based SLR automation (Alshami et al., 2023; Dunn et al., 2022; Gartlehner et al., 2024; Guo et al., 2024; Khraisha et al., 2024; Kohandel Gargari et al., 2024; Landschaft et al., 2024; Li et al., 2024; Matsui et al., 2024; Tran et al., 2024; Wang Z. et al., 2025). The average time taken for the human reviewers to screen each study may not be representative of other SLRs, as cost-effectiveness analyses have a standardized outcome reporting format; ISLaR’s relative time savings may thus be greater for other study types. The low specificity score in the abstract screening task was due to the intentional use of less stringent inclusion and exclusion criteria at this stage compared to the full-text screening stage; as expected, this score improved substantially in the full-text screening task. However, even in this second, more stringent screen, ISLaR’s specificity score was lower than its sensitivity score. This finding is consistent with several other studies (Alshami et al., 2023; Guo et al., 2024; Khraisha et al., 2024; Kohandel Gargari et al., 2024; Matsui et al., 2024; Tran et al., 2024), although a smaller number of studies have reported both high sensitivity and high specificity (Landschaft et al., 2024; Li et al., 2024). Since most SLRs ultimately exclude a substantial number of potentially eligible articles (Sampson et al., 2011; Wang et al., 2020; Yaffe et al., 2012), the use of highly sensitive LLM tools to rapidly screen out obviously irrelevant articles would in itself substantially reduce researchers’ workloads while ensuring that almost all relevant articles are retained for detailed manual review. ISLaR and other LLM-based tools could also find similar applications in first-pass screening of newly published articles that may be eligible for inclusion in living SLRs (Elliott et al., 2014; Wijkstra et al., 2021). Future avenues of development could include exploration of article selection formats other than the current binary include/exclude decision, such as numerical relevance scores and dynamic article ranking systems similar to those included in some NLP-based automation tools (Akinseloyin et al., 2024; Bolaños et al., 2024; Oude Wolcherink et al., 2023; van Dijk et al., 2023).

While ISLaR performed well in extracting data from the texts of included studies, its performance in extracting data from tables was substantially weaker and, in our opinion, not yet adequate for use in SLRs. This pattern was reflected across multiple performance metrics. For example, F2 and MCC values were high for text-based extractions but dropped significantly for table-based extractions. Further, ISLaR’s table data extraction errors required additional manual effort to understand and resolve compared to its text data extraction errors, due to frequent occurrences of missing contextual information (such as study cohort), formatting errors in the extracted data, and references to the wrong table number in the data extraction notes. Wang Z. et al. (2025) reported a potentially related phenomenon whereby their LLM-based tool performed best at accurately extracting study design variables (which would be expected to occur in study texts), and worst at extracting outcomes and other numerical data (which would be more likely to appear in study tables). It would thus be advisable to maintain a human-in-the-loop model at least until the performance of LLMs on data extraction tasks improves. Future studies should assess the additional reviewer time needed to correct mistakes introduced by ISLaR or other LLMs in human-in-the-loop models. Other data extraction errors could potentially be mitigated by fine-tuning ISLaR’s underlying LLM to improve its performance on biomedical texts, or using a different LLM that has already been optimized for this purpose (Landschaft et al., 2024; Luo et al., 2024; Robinson et al., 2023; Wu et al., 2024). Prompt development is also a critical aspect of optimizing LLM performance, as the clarity and consistency of user-provided inclusion and exclusion criteria strongly influence system performance and the risk of bias.

Despite the issues with data extraction from tables, we observed several cases where ISLaR was able to correctly extract data that were marked as not present by one or more human reviewers. These human errors were likely due to reviewer fatigue, highlighting a potential benefit of automation in terms of better reproducibility and lower risk of bias (Alshami et al., 2023; Guo et al., 2024; Matsui et al., 2024). In particular, ISLaR’s ability to identify correct values that human reviewers missed highlights its notable potential to complement human effort and reduce the impact of fatigue or oversight. Even modest reductions in fatigue-related mistakes could meaningfully improve the consistency and reliability of SLR outputs, especially for tasks that require sustained attention to repetitive details. However, the reproducibility of LLMs can sometimes be affected by changes in the underlying model that are not transparently documented and available to users, and which can change the outputs generated in response to the same prompt over time (Chen et al., 2023; Gartlehner et al., 2024; O’Connor et al., 2024; Syriani et al., 2023). Reproducibility was not formally assessed in the current study, but we note that when asked to reassess its full-text article exclusion decisions, ISLaR changed its response for 3 of 36 articles. Conducting all tasks for a given stage of an SLR in a single session, and/or replicating tasks to obtain a consensus, may help to mitigate these issues.

Although we have demonstrated the potential utility of ISLaR in rapidly performing SLR tasks to ease researcher workload, there are many challenges and barriers that must be addressed before AI tools can be routinely used in practice (Bolaños et al., 2024; de la Torre-López et al., 2023; Doyal et al., 2023; O’Connor et al., 2019; van Altena et al., 2019). Researchers’ current lack of trust in the accuracy and quality of SLR automation tools has been identified as a major barrier to uptake, and transparency from the developers and users of these tools has been suggested as a means to overcome this challenge (Bolaños et al., 2024; de la Torre-López et al., 2023; Gates et al., 2019; O’Connor et al., 2019; van Altena et al., 2019). The ability of LLMs to document the rationale behind each screening and data extraction decision is a major advantage over earlier AI approaches, improving transparency and aiding in the interpretation of the models’ outputs, as well as informing the future development of models and prompts for better performance (Bolaños et al., 2024; de la Torre-López et al., 2023). For example, the decision rationales provided by ISLaR highlight the platform’s difficulty in distinguishing certain study variables from background information; others have reported similar problems (Landschaft et al., 2024). However, some of ISLaR’s decision rationales—particularly secondary exclusion reasons for studies—were more difficult to interpret. Future studies will explore the impact of limiting ISLaR’s rationale output to a single reason to improve interpretability and, ultimately, transparency. Additionally, further work should explore how the metrics calculated here compare to more classical ML baselines for SLRs.

Our work to make ISLaR’s decisions transparent to users also aligns with global efforts to update SLR best-practice guidelines to include advice on the use of automation tools. For example, the 2020 update of the Preferred Reporting Items for Systematic reviews and Meta-Analyses (PRISMA) checklist now prompts SLR authors to document any automation tools used in article selection, data extraction, and risk of bias assessments, as well as the study inclusion and exclusion decisions made by these tools (Page et al., 2021). Similarly, the Professional Society for Health Economics and Outcomes Research (ISPOR) Criteria for Cost (-Effectiveness) Review Outcomes (CiCERO) checklist and guidance document stress that any automation tools used in an SLR should be reported transparently (Mandrik et al., 2021). In its complementary efforts to advance the development of and uptake of AI tools for evidence synthesis, the International Collaboration for the Automation of Systematic Reviews (ICASR) also emphasizes the importance of transparent reporting of performance metrics (Beller et al., 2018; O’Connor et al., 2024). More recently, ICASR has published guidance on the responsible use of AI in evidence synthesis (Responsible AI in Systematic Evidence synthesis, or RAISE), underscoring the need for transparency in reporting and the careful application of AI to ensure responsible use (Thomas et al., 2025).

### 4.1 Strengths and limitations

Our choice of case-study SLR was a major strength of the current analysis as it enabled us to perform a rigorous and comprehensive assessment of ISLaR’s performance in a prospective comparison to expert human researchers. This analysis included assessment of the reasons for ISLaR’s errors as well as areas of particular strength for both ISLaR and human reviewers, to improve transparency and inform the future development and use of the platform.

However, the current study has some known limitations. We used stringent search queries that returned relatively few articles for screening; ISLaR might perform differently if working from a broader search query that retrieves more ineligible studies. For example, ChatGPT-4 has been reported to have higher accuracy when screening more imbalanced datasets (i.e., groups of articles that included a higher proportion of irrelevant studies) (Khraisha et al., 2024). In addition, our data extraction process used only the main texts of included articles; future development should focus on allowing ISLaR to assess and extract data from supplementary materials, as well as gray literature, which may contain additional relevant information (Lawrence et al., 2014; Mandrik et al., 2021; Paez, 2017). Finally, the performance of ISLaR in the case-study SLR suggests that the platform is not yet ready for fully autonomous use and requires a “human-in-the-loop” model. However, we have not yet been able to identify a means of highlighting which of ISLaR’s decisions are most likely to be incorrect and to require additional scrutiny by a human reviewer. This is an important area for improvement.

In conclusion, our analysis of ISLaR’s performance in a prospective SLR demonstrates that the platform has potential to reduce the workload involved in an SLR, particularly during the article screening stage and parts of the data extraction process. It could also be used for the ongoing screening of newly published articles for potential inclusion in living SLRs. However, the platform is not yet ready for autonomous use, and the current version requires oversight from expert researchers.

## Data Availability

The original contributions presented in the study are included in the article/Supplementary material, further inquiries can be directed to the corresponding author.
