# Supplementary material for: Analysis of article screening and data extraction performance by an AI systematic literature review platform
Source: Front Artif Intell. 2025 Nov 20;8:1662202. doi: 10.3389/frai.2025.1662202 (PMC12676488; doi:10.3389/frai.2025.1662202)
Supplement: Supplementary file 1 [file Table_1.DOCX]

Supplementary Material

# Supplementary Data

Supplementary Table 1. Literature search strategy and results

| Database | Strategy | Final results |
| --- | --- | --- |
| PubMed | (("pneumococcal vaccines"[MeSH Terms] OR ("pneumococcal"[All Fields] AND "vaccines"[All Fields]) OR "pneumococcal vaccines"[All Fields] OR ("pneumococcal"[All Fields] AND "vaccine"[All Fields]) OR "pneumococcal vaccine"[All Fields]) AND ("cost effectiveness analysis"[MeSH Terms] OR ("cost effectiveness"[All Fields] AND "analysis"[All Fields]) OR "cost effectiveness analysis"[All Fields] OR ("cost"[All Fields] AND "effectiveness"[All Fields] AND "analysis"[All Fields]) OR "cost effectiveness analysis"[All Fields]) AND ("adult"[MeSH Terms] OR "adult"[All Fields] OR "adults"[All Fields] OR "adults"[All Fields])) AND ((excludepreprints[Filter]) AND (2011/1/1:2023/1/1[pdat]) AND (english[Filter])) | 145 |
| EMBASE | ('Streptococcus pneumoniae'/exp AND 'vaccine'/exp) OR ('Streptococcus pneumoniae'/exp AND 'immuni*ation'/exp) AND ('economic evaluation'/exp OR 'Markov chain'/exp) AND [english]/lim AND ([adult]/lim OR [aged]/lim OR [very elderly]/lim) AND ([embase]/lim OR [medline]/lim) AND [2011-2023]/py AND ([article]/lim OR [article in press]/lim OR [data papers]/lim OR [review]/lim) | 37 |

^A^ This search query was run on July 18, 2024.

**Supplementary Table 2. Data extraction fields**

| Element | Definition | Example text span ^A^ |
| --- | --- | --- |
| Text-based fields |  |  |
| Fields relevant to entire study | | |
| Primary objective | The main goal of the study, typically related to evaluating the cost effectiveness of pneumococcal vaccination | ‘To evaluate the cost effectiveness of the 13-valent pneumococcal conjugate vaccine compared to the 23-valent pneumococcal polysaccharide vaccine in preventing invasive pneumococcal disease in adults’ |
| Competing strategies | Detailed description of the vaccination strategies being compared, often formatted as ‘X versus Y’ | ‘The study compares the strategy of no vaccination, the use of the 13-valent pneumococcal conjugate vaccine (PCV13), and the 23-valent pneumococcal polysaccharide vaccine (PPSV23) in preventing pneumococcal diseases’ |
| Model type | The type of economic model used to conduct the cost-effectiveness analysis. Examples include Markov models and decision-analytic models | ‘Markov model’ or ‘Decision tree model’ |
| Cycle length | The length of each cycle or stage in the economic model, typically measured in years | ‘Model cycle length of 1 year’ |
| Time horizon | The period over which costs and benefits are evaluated, typically measured in years | ‘A time horizon of 20 years’ |
| Payer perspective | The perspective from which costs and benefits are analyzed, such as societal or healthcare payer perspective | ‘Healthcare payer perspective’ |
| Monetary denomination | The denomination of money used when estimating costs | ‘Costs were estimated in USD’ |
| Year of denomination | The year that costs were estimated | ‘The cost base year was 2006’ |
| Discount | The discount rate applied to costs | ‘The costs and effectiveness were discounted 3% per year’ |
| Sensitivity analysis | What was the sensitivity analysis conducted | ‘The likelihood varied widely (from 5% to 95%) in sensitivity analyses’ |
| Study limitations | The limitations of the study design and results | ‘The study was limited to analysis of adults and did not include adjustments for herd immunity due to vaccination in children’ |
| Generalizability | Extent to which the study’s findings can be generalized to other populations | ‘The results of this study may not be generalizable to other populations or settings’ |
| Conclusion | Study conclusion | ‘The current analyses suggest that PCV13 should prevent more pneumococcal disease and be economically favorable when compared to the currently recommended PCV7’ |
| Conflict of interest | Author conflict of interest or financial disclosure | ‘Dr. Smith has no current conflicts of interest but within the previous 3 years, had research grants from Merck & Co., Inc., Rahway, NJ, USA.’ |
| Fields relevant to specific study cohort(s) | | |
| IPD definition | How IPD was defined in the study | ‘IPD is defined as pneumococcal bacteremia or meningitis’ |
| Total cases included | The specific disease states included in the total case count | ‘Total cases include invasive pneumococcal disease, bacteremia, and meningitis’ |
| Total deaths included | The specific disease states included in the total death count | ‘Total deaths include deaths due to invasive pneumococcal disease, bacteremia, and meningitis’ |
| Total costs included | The components included in the total cost calculation, such as direct and indirect costs | ‘Total costs include vaccine acquisition costs, administration costs, and healthcare utilization costs’ |
| Total medical costs included | The components included in the total medical cost calculation, such as hospitalization and medication costs | ‘Total medical costs include hospitalization costs, outpatient visit costs, and medication costs’ |
| Reference group | The group receiving the standard of care vaccine strategy or no vaccination | ‘PCV13 as the reference group’ |
| Comparison group | The vaccine strategy being compared to the reference group, or the standard of care | ‘PPSV23 as the comparison group’ |
| Age group | Specific age range of population studied | ‘Adults aged 65 and older’ |
| Risk group | Specific population included in study and at increased risk for PD | ‘Individuals with chronic heart, lung, or liver disease’ |
| Table-based fields |  |  |
| Fields relevant to specific study cohort(s) | | |
| Bacteremia cases ^B^ | Number of cases of bacteremic / septic PD | ‘Bacteremia cases for PCV13 group is 2,000’ |
| IPD cases ^B^ | Number of cases of IPD | ‘IPD cases for PCV13 group is 3,000’ |
| Meningitis cases ^B^ | Number of cases of pneumococcal meningitis | ‘Meningitis cases for PCV13 group is 1,000’ |
| NBPP hospitalized cases ^B^ | Number of hospitalized cases of NBPP | ‘Hospitalized NBPP cases for PCV13 group is 6,000’ |
| NBPP outpatient cases ^B^ | Number of outpatient cases of NBPP (including ER and primary care visits) | ‘Outpatient NBPP cases for PCV13 group is 5,000’ |
| Total cases ^B^ | The total number of PD cases | ‘Total case difference is -2,000 (PCV13 - PPSV23)’ |
| Bacteremia deaths ^B^ | Number of deaths due to bacteremic / septic PD | ‘Bacteremia deaths difference is -20 (PCV13-PPSV23)’ |
| IPD deaths ^B^ | Number of deaths due to IPD | ‘IPD deaths difference is -50 (PCV13 - PPSV23)’ |
| Meningitis deaths ^B^ | Number of deaths due to pneumococcal meningitis | ‘Meningitis deaths difference is -10 (PCV13 - PPSV23)’ |
| NBPP hospitalized deaths ^B^ | Number of deaths due to hospitalized NBPP | ‘Hospitalized NBPP deaths difference is -30 (PCV13 - PPSV23)’ |
| Total deaths ^B^ | The total number of deaths due to pneumococcal infection | ‘Total deaths difference is -50 (PCV13 - PPSV23)’ |
| LY ^B^ | Total LYs gained or lost by each group due to vaccination or illness | ‘Life year difference is 2,000 (PCV13 - PPSV23)’ |
| QALY ^B^ | Total QALYs gained or lost by each group due to vaccination or illness | ‘QALY difference is 2,000 (PCV13 - PPSV23)’ |
| Total medical cost ^B^ | The total medical costs incurred by the vaccination strategy, excluding non-medical costs | ‘Total medical cost for PCV13 group is $400 million’ |
| Non-medical cost ^B^ | Non-medical or societal costs associated with the vaccination strategy | ‘Non-medical cost difference is $0’ |
| Total cost ^B^ | The total cost incurred by the vaccination strategy, typically including both healthcare and indirect costs | ‘Total cost for PCV13 group is $500 million’ |
| ICER (LY) | Difference between two strategies in the cost per additional LY gained | ‘LY ICER is $20,000 per life year gained’ |
| ICER (QALY) | Difference between two strategies in the cost per additional QALY gained | ‘QALY ICER is $25,000 per QALY gained’ |

ER, emergency room; IPD, invasive pneumococcal disease; LY, life year; NBPP, non-bacteremic pneumococcal pneumonia; QALY, quality-adjusted life year; PCV, pneumococcal conjugate vaccine; PD, pneumococcal disease; PPSV, pneumococcal polysaccharide vaccine; USD, United States dollars.

^A^ Written by the study’s authors and provided to the AI tool development team to guide prompt development.

^B^ Stated as the value for the reference group, comparison group, or the difference between groups, stated for example as the number of cases/deaths averted by the vaccine intervention of interest.

Supplementary Table 3. Instructions used in the LLM prompt to guide the screening process

| Abstract screening |
| --- |
| To help make the eligibility decision, please carefully consider the following instruction:  Does any one of the exclusion criteria under Population, Intervention/Comparators, Outcomes, or Other categories perfectly match given the abstract title and text? If the answer is clearly "Yes", then "Exclude" the abstract, otherwise "Include" the abstract.  Note: If there is insufficient information in the abstract to decide whether to include or exclude the abstract, please consider to "Include" the abstract. |
| Full text screening |
| To help make the eligibility decision, please carefully consider the following steps one by one:  1. The article's study design and publication type value are -- {study_design_publication_type_extracted_by_separate_prompt}. Does any one of the exclusion criteria under Population, Intervention/Comparators, Outcomes, or Other categories perfectly match given the article's full text and the article's study design and publication types?  2. If answer to question 1 is clearly "Yes", only then "Exclude" the article, otherwise, consider the following five questions related to inclusion criteria:  --> 2.1 Does each and every inclusion criterion under Population category strictly match given the article?  --> 2.2. Does each and every inclusion criterion under Intervention/Comparators category strictly match given the article?  --> 2.3. Does each and every inclusion criterion under Outcomes category strictly match given the article?  --> 2.4. The article's study design and publication type value are -- {study_design_publication_type_extracted_by_previous_prompt}. Only in case any study design or publication type criteria is mentioned under the Inclusion Criteria, consider this question, otherwise please do not consider this question. The question is - Does the article's study design and publication type value belong to any one of the study design and publication type categories mentioned under the inclusion criteria?  --> 2.5. Does each and every inclusion criterion under Other category strictly match given the article?  3. If answer to question 2.4 is "No", then "Exclude" the article. Otherwise, if answers to all five questions above under step 2 (that is, 2.1, 2.2, 2.3, 2.4, and 2.5) are clearly "Yes", then "Include" the article.  For all other scenarios, label as "Include - (not very confident)".  Note: Classify an article as "Include - (not very confident)" only when you are not very confident about including an article based on the information in the inclusion and exclusion criteria points. |

Supplementary Table 4. Cohen’s Kappa comparing human–human and human–ISLaR screening results

| Stage | Cohen’s Kappa | |
| --- | --- | --- |
|  | Human 1–Human 2 | Human (combined)–ISLaR |
| Abstract screening | 0.65 | 0.50 |
| Full-text screening | 0.75 | 0.73 |

Supplementary Table 5. Categorization of the full set of 160 studies at the abstract and full-text screening stages

| Citation | Title | Screening result ^A^ | |
| --- | --- | --- | --- |
|  |  | Abstract | Full text |
| Akazawa 2014 (1) | Considering economic analyses in the revision of the preventive vaccination law: a new direction for health policy-making in Japan? | TP | TN |
| Akin 2011 (2) | Cost of pneumococcal infections and cost-effectiveness analysis of pneumococcal vaccination at risk adults and elderly in Turkey | TP | TP |
| Aljunid 2011 (3) | Impact of routine PCV7 (Prevenar) vaccination of infants on the clinical and economic burden of pneumococcal disease in Malaysia | FP | TN |
| Ansaldi 2020 (4) | Estimating the clinical and economic impact of switching from the 13-valent pneumococcal conjugate vaccine (PCV13) to the 10-valent pneumococcal conjugate vaccine (PCV10) in Italy | TP | TN |
| Astengo 2022 (5) | Incidence of Hospitalisation and Emergency Department Visits for Pneumococcal Disease in Children, Adolescents, and Adults in Liguria, Italy: A Retrospective Analysis from 2012–2018 | TN | NA |
| Atwood 2018 (6) | Cost-effectiveness of alternative strategies for use of 13-valent pneumococcal conjugate vaccine (PCV13) in Canadian adults | TP | TP |
| Bar-Zeev 2015 (7) | Methods and challenges in measuring the impact of national pneumococcal and rotavirus vaccine introduction on morbidity and mortality in Malawi | TP | TN |
| Berical 2016 (8) | Pneumococcal Vaccination Strategies. An Update and Perspective | FP | TN |
| Betsch 2018 (9) | Increasing influenza and pneumococcal vaccine uptake in the elderly: study protocol for the multi-methods prospective intervention study Vaccination60 | FN | TN |
| Biagini 2018 (10) | Cost-Utility Study of PCV13 Versus PPSV23 in Adults in Chile | TP | FP |
| Birck 2021 (11) | Health economic evaluation of introducing a PPSV23-based vaccination programme to adults aged 65 and above, and an extension to the 60-64 age group in Denmark | TP | TP |
| Blank 2012 (12) | Cost-effectiveness of 13-valent pneumococcal conjugate vaccine in Switzerland | TP | TN |
| Blasi 2012 (13) | Understanding the burden of pneumococcal disease in adults | TP | TN |
| Blommaert 2016 (14) | The cost-effectiveness of pneumococcal vaccination in healthy adults over 50: An exploration of influential factors for Belgium | TP | TP |
| Boccalini 2013 (15) | Cost-effectiveness of new adult pneumococcal vaccination strategies in Italy | TP | TP |
| Boccalini 2017 (16) | Economic studies applied to vaccines against invasive diseases: An updated budget impact analysis of age-based pneumococcal vaccination strategies in the elderly in Italy | TP | FN |
| Bolaños-Díaz 2022 (17) | Cost-effectiveness of the 13-valent pneumococcal conjugate vaccine compared to the 10-valent vaccine in children: predictive analysis in the Ecuadorian context | FN | TN |
| Buchwald 2021 (18) | Response to Shami et al. 'Evaluating the cost-effectiveness of a sequential pneumococcal vaccination compared to single dose vaccination strategy for adults in Hong Kong' (Hum Vacc Immunother 2020) | TP | TN |
| Caldwell 2015 (19) | The health and economic impact of vaccination with 7-valent pneumococcal vaccine (PCV7) during an annual influenza epidemic and influenza pandemic in China | FP | TN |
| Castaneda-Orjuela 2011 (20) | Cost-effectiveness of the introduction of the pneumococcal polysaccharide vaccine in elderly Colombian population | TP | TP |
| Chaiyakunapruk 2022 (21) | Public Health Value of a Hypothetical Pneumococcal Conjugate Vaccine (PCV) Introduction: A Case Study | TN | NA |
| CDC 2012 (22) | Use of 13-valent pneumococcal conjugate vaccine and 23-valent pneumococcal polysaccharide vaccine for adults with immunocompromising conditions: Recommendations of the Advisory Committee on Immunization Practices (ACIP) | TP | TN |
| Chen 2014 (23) | Cost-effectiveness of pneumococcal vaccines for adults in the United States | TP | TP |
| Chen 2018a (24) | Evolution over time in the cost-effectiveness of pneumococcal conjugate vaccine (PCV13) in older Australians due to herd protection from infant vaccination | TP | TP |
| Chen 2018b (25) | Retrospective cost-effectiveness of the 23-valent pneumococcal polysaccharide vaccination program in Australia | TP | FN |
| Chen 2018c (26) | The role of timeliness in the cost-effectiveness of older adult vaccination: A case study of pneumococcal conjugate vaccine in Australia | TP | TP |
| Chen 2021 (27) | Cost-effectiveness of dual influenza and pneumococcal vaccination among the elderly in Shenzhen, China | TP | TP |
| Cho 2013 (28) | Cost-effectiveness of administering 13-valent pneumococcal conjugate vaccine in addition to 23-valent pneumococcal polysaccharide vaccine to adults with immunocompromising conditions | TP | TP |
| Choi 2018 (29) | Cost-effectiveness analysis of 13-valent pneumococcal conjugate vaccine versus 23-valent pneumococcal polysaccharide vaccine in an adult population in South Korea | TP | TP |
| Christie 2011 (30) | Long-term outcomes of pneumococcal meningitis in childhood and adolescence | FN | TN |
| Dao 2018 (31) | Socioeconomic burden of community-acquired pneumonia associated hospitalizations among Vietnamese patients: A prospective, incidence-based study | TN | NA |
| Deb 2022 (32) | Cost-effectiveness of the 15-valent pneumococcal conjugate vaccine for high-risk adults in Switzerland | TP | TP |
| de Soarez 2015 (33) | Cost-Effectiveness Analysis of Universal Vaccination of Adults Aged 60 Years with 23-Valent Pneumococcal Polysaccharide Vaccine versus Current Practice in Brazil | FN | TP |
| de Vries 2021 (34) | Don't forget about the future: The impact of including future costs on the cost-effectiveness of adult pneumococcal conjugate vaccination with PCV13 in the Netherlands | TP | TP |
| Díez-Domingo 2011 (35) | Pharmacoeconomic assessment of implementing a universal PCV-13 vaccination programme in the Valencian public health system (Spain) | TP | TN |
| Dilokthornsakul 2019 (36) | An updated cost-effectiveness analysis of pneumococcal conjugate vaccine among children in Thailand | FP | TN |
| Ding 2022 (37) | The cost-effectiveness of starting 23-valent pneumococcal polysaccharide vaccine and influenza vaccination at 50 vs. 65 years: A comparative modelling study | TP | TP |
| Dirmesropian 2015 (38) | A review of economic evaluations of 13-valent pneumococcal conjugate vaccine (PCV13) in adults and the elderly | TN | NA |
| Dirmesropian 2017 (39) | Cost-effectiveness of 13-valent pneumococcal conjugate vaccine (PCV13) in older Australians | TP | TP |
| Dominguez 2013 (40) | Effectiveness of vaccination with 23-valent pneumococcal polysaccharide vaccine in preventing hospitalization with laboratory confirmed influenza during the 2009-2010 and 2010-2011 seasons | TP | TN |
| Dorji 2018 (41) | Towards the introduction of pneumococcal conjugate vaccines in Bhutan: A cost-utility analysis to determine the optimal policy option | TP | FP |
| Earnshaw 2012 (42) | Cost-effectiveness of 2 + 1 dosing of 13-valent and 10-valent pneumococcal conjugate vaccines in Canada | FP | TN |
| Eilers 2013 (43) | Assessment of vaccine candidates for persons aged 50 and older: a review | TN | NA |
| Eythorsson 2021 (44) | The impact and cost-effectiveness of introducing the 10-valent pneumococcal conjugate vaccine into the paediatric immunisation programme in Iceland-A population-based time series analysis | FN | TN |
| Feemster 2022 (45) | Response to Igarashi, et al, cost-effectiveness analysis for PCV13 in adults 60 years and over with underlying medical conditions which put them at an elevated risk of pneumococcal disease in Japan | TP | FP |
| Feldman 2020 (46) | The cost-effectiveness of using pneumococcal conjugate vaccine (PCV13) versus pneumococcal polysaccharide vaccine (PPSV23), in South African adults | TP | TP |
| Gaillat 2013 (47) | Compared effectiveness of the 7-valent pneumococcal conjugate vaccine in children with the 13-valent vaccine in adults | TP | TN |
| Gamil 2021 (48) | Pneumococcal disease in Thailand | FP | TN |
| Giglio 2022 (49) | Cost-Effectiveness of Pneumococcal Vaccines for Adults Aged 65 Years and Older in Argentina | TP | TP |
| Gil de Miguel 2022 (50) | Direct Medical Costs of Four Vaccine-Preventable Infectious Diseases in Older Adults in Spain | TP | TN |
| Gilchrest 2012 (51) | Benefits and effectiveness of administering pneumococcal polysaccharide vaccine with seasonal influenza vaccine: an approach for policymakers | FP | TN |
| Gouveia 2017 (52) | Cost-effectiveness of the 13-valent Pneumococcal Conjugate Vaccine in Children in Portugal | FP | TN |
| Gouveia 2019 (53) | Cost-effectiveness of the 13-valent pneumococcal conjugate vaccine in adults in Portugal versus "no vaccination" and versus vaccination with the 23-valent pneumococcal polysaccharide vaccine | TP | TP |
| Grzesiowski 2012 (54) | Cost-effectiveness of polysaccharide pneumococcal vaccination in people aged 65 and above in Poland | TP | TP |
| Haasis 2015 (55) | Do Pneumococcal Conjugate Vaccines Represent Good Value for Money in a Lower-Middle Income Country? A Cost-Utility Analysis in the Philippines | TP | FP |
| Hall 2021 (56) | COVID-19 vaccine coverage in health-care workers in England and effectiveness of BNT162b2 mRNA vaccine against infection (SIREN): a prospective, multicentre, cohort study | TN | NA |
| Heo 2017 (57) | Cost-effectiveness of pneumococcal vaccination strategies for the elderly in Korea | TP | TP |
| Hoshi 2015 (58) | Economic Evaluation of Immunisation Programme of 23-Valent Pneumococcal Polysaccharide Vaccine and the Inclusion of 13-Valent Pneumococcal Conjugate Vaccine in the List for Single-Dose Subsidy to the Elderly in Japan | TP | TP |
| Hoshi 2022 (59) | Cost-effectiveness analyses of 15- and 20-valent pneumococcal conjugate vaccines for Japanese elderly | TP | TP |
| Igarashi 2021 (60) | Cost-effectiveness analysis for PCV13 in adults 60 years and over with underlying medical conditions which put them at an elevated risk of pneumococcal disease in Japan | TP | TP |
| Igarashi 2022 (61) | Response to Feemster KA et al. letter to the editor on Cost-effectiveness analysis for PCV13 in adults 60 years and over with underlying medical conditions which put them at an elevated risk of pneumococcal disease in Japan | TP | TN |
| Ignatova 2021 (62) | Comparative effectiveness of pneumococcal vaccination with PPV23 and PCV13 in COPD patients over a 5-year follow-up cohort study | TP | TN |
| Ishigami 2019 (63) | Cost-effectiveness of Pneumococcal Vaccination Among Patients With CKD in the United States | TP | TP |
| Jiang 2012 (64) | Cost-effectiveness of vaccinating adults with the 23-valent pneumococcal polysaccharide vaccine (PPV23) in Germany | TP | TP |
| Jiang 2014 (65) | Cost-effectiveness of vaccinating the elderly and at-risk adults with the 23-valent pneumococcal polysaccharide vaccine or 13-valent pneumococcal conjugate vaccine in the UK | TP | TP |
| Jiang 2018 (66) | A cost-effectiveness analysis of revaccination and catch-up strategies with the 23-valent pneumococcal polysaccharide vaccine (PPV23) in older adults in Japan | TP | TP |
| José, 2017 (67) | Adult pneumococcal vaccination: Advances, impact, and unmet needs | FP | TN |
| Klok 2013 (68) | Cost-effectiveness of a 10- versus 13-valent pneumococcal conjugate vaccine in Denmark and Sweden | FP | TN |
| Knerer 2012 (69) | Health and economic impact of PHiD-CV in Canada and the UK: a Markov modelling exercise | TP | TN |
| Kobayashi 2021 (70) | Cost-effectiveness of implementing 13-valent pneumococcal conjugate vaccine for U.S. adults aged 19 years and older with underlying conditions | TP | TP |
| Kuchenbecker 2018 (71) | Estimating the cost-effectiveness of a sequential pneumococcal vaccination program for adults in Germany | TP | TP |
| Kuhlman 2017 (72) | Modeling the cost-effectiveness of infant vaccination with pneumococcal conjugate vaccines in Germany | TN | NA |
| Kulpeng 2013 (73) | Cost-utility analysis of 10- and 13-valent pneumococcal conjugate vaccines: protection at what price in the Thai context? | TP | FP |
| Lawrence 2020 (74) | Effectiveness of the 23-valent pneumococcal polysaccharide vaccine against vaccine serotype pneumococcal pneumonia in adults: A case-control test-negative design study | TP | TN |
| Leidner 2019 (75) | Cost-effectiveness of adult vaccinations: A systematic review | TN | NA |
| Liguori 2014 (76) | Adult immunization with 13-valent pneumococcal vaccine in Campania region, South Italy: an economic evaluation | TP | FN |
| Lin 2013 (77) | Cost-effectiveness of pneumococcal and influenza vaccination standing order programs | TP | TP |
| Mangen 2015 (78) | Cost-effectiveness of adult pneumococcal conjugate vaccination in the Netherlands | TP | TP |
| Marbaix 2018 (79) | Cost-effectiveness of PCV13 vaccination in Belgian adults aged 65-84 years at elevated risk of pneumococcal infection | TP | TP |
| Mauskopf 2022 (80) | Economic Evaluation of Vaccination Programs: A Guide for Selecting Modeling Approaches | TN | NA |
| McGarry 2013 (81) | Impact of 13-valent pneumococcal conjugate vaccine (PCV13) in a pandemic similar to the 2009 H1N1 in the United States | TP | TN |
| Mendes 2022 (82) | Cost-effectiveness of using a 20-valent pneumococcal conjugate vaccine to directly protect adults in England at elevated risk of pneumococcal disease | TP | TP |
| Michaelidis 2013 (83) | Cost-effectiveness of a program to eliminate disparities in pneumococcal vaccination rates in elderly minority populations: an exploratory analysis | TP | TP |
| Migdal 2019 (84) | Time Cost of Standardized Nursing Screens in the Emergency Department | FN | TN |
| Musher 2016 (85) | Why the recent ACIP recommendations regarding conjugate pneumococcal vaccine in adults may be irrelevant | TP | TN |
| Nakamura 2011 (86) | Cost effectiveness of child pneumococcal conjugate vaccination in middle-income countries | FP | TN |
| Namkoong 2016 (87) | Theory and strategy for Pneumococcal vaccines in the elderly | TP | TN |
| Naucler 2017 (88) | Comparison of the Impact of Pneumococcal Conjugate Vaccine 10 or Pneumococcal Conjugate Vaccine 13 on Invasive Pneumococcal Disease in Equivalent Populations | TP | TN |
| Neto 2011 (89) | Cost-effectiveness analysis of pneumococcal polysaccharide vaccination from age 60 in São Paulo State, Brazil | TP | TP |
| Newall 2016a (90) | Retrospective economic evaluation of childhood 7-valent pneumococcal conjugate vaccination in Australia: Uncertain herd impact on pneumonia critical | TP | TN |
| Newall 2016b (91) | What do we know about the cost-effectiveness of pneumococcal conjugate vaccination in older adults? | TP | TN |
| Newall 2019 (92) | Reply to letter: Retrospective cost-effectiveness of the 23-valent pneumococcal polysaccharide vaccination program in Australia | TP | FN |
| Nicholson 2014 (93) | Randomised controlled trial and health economic evaluation of the impact of diagnostic testing for influenza, respiratory syncytial virus and Streptococcus pneumoniae infection on the management of acute admissions in the elderly and high-risk 18- to 64-year-olds | TN | NA |
| Nishikawa 2018 (94) | Systematic review of economic evaluations of the 23-valent pneumococcal polysaccharide vaccine (PPV23) in individuals 60 years of age or older | TN | NA |
| Nymark 2022 (95) | Cost-utility analysis of the universal pneumococcal vaccination programme for older adults in Norway | TP | TP |
| Olsen 2022 (96) | Cost-effectiveness of 20-valent pneumococcal conjugate vaccine in Denmark compared with PPV23 | TP | TP |
| Ordonez 2014 (97) | Cost-effectiveness analysis of pneumococcal conjugate vaccine 13-valent in older adults in Colombia | TP | TP |
| Perdrizet 2021 (98) | Cost-effectiveness analysis of replacing the 10-valent pneumococcal conjugate vaccine (PCV10) with the 13-valent pneumococcal conjugate vaccine (PCV13) in Brazil infants | FP | TN |
| Piralam 2015 (99) | Incidence of pneumococcal pneumonia among adults in rural Thailand, 2006-2011: Implications for pneumococcal vaccine considerations | TP | TN |
| Pircon 2018 (100) | The choice of analytical methodology can alter conclusions regarding herd effects of paediatric pneumococcal vaccination programmes | FN | TN |
| Pitsiou 2011 (101) | Pneumococcal vaccination in adults: does it really work? | TP | TN |
| Pletz 2015 (102) | Pneumococcal conjugate vaccine for adults: "it's tough to make predictions, …" | TP | TN |
| Plosker 2015 (103) | 13-Valent Pneumococcal Conjugate Vaccine: A Review of Its Use in Adults | FP | NA ^B^ |
| Polistena 2022 (104) | Cost-Effectiveness of Vaccination with the 20-Valent Pneumococcal Conjugate Vaccine in the Italian Adult Population | TP | TP |
| Porchia 2017 (105) | Evaluating the costs and benefits of pneumococcal vaccination in adults | FP | TN |
| Pradas 2013 (106) | Budget impact analysis of a pneumococcal vaccination programme in the 65-year-old Spanish cohort using a dynamic model | TP | TN |
| Pugh 2020 (107) | Estimating the Impact of Switching from a Lower to Higher Valent Pneumococcal Conjugate Vaccine in Colombia, Finland, and The Netherlands: A Cost-Effectiveness Analysis | FN | TN |
| Ren 2021 (108) | Pneumococcal polysaccharide vaccine is a cost saving strategy for prevention of acute coronary syndrome | TP | FN |
| Robberstad 2011 (109) | Economic evaluation of second generation pneumococcal conjugate vaccines in Norway | TP | FP |
| Rodgers 2016 (110) | Surveillance of the impact of pneumococcal conjugate vaccines in developing countries | TP | TN |
| Rodriguez Gonzalez-Moro 2016 (111) | Cost Effectiveness of the 13-Valent Pneumococcal Conjugate Vaccination Program in Chronic Obstructive Pulmonary Disease Patients Aged 50+ Years in Spain | TP | TP |
| Rozenbaum 2012 (112) | Vaccination of risk groups in England using the 13 valent pneumococcal conjugate vaccine: economic analysis | TP | TN |
| Sanduzzi 2019 (113) | Impact of 13valent vaccine for prevention of pneumococcal diseases in children and adults at risk: Possible scenarios in Campania region | FN | TN |
| Sehatzadeh 2012 (114) | Influenza and pneumococcal vaccinations for patients with chronic obstructive pulmonary disease (COPD): an evidence-based review | TN | NA |
| Sevilla 2020 (115) | Indirect costs of adult pneumococcal disease and the productivity-based rate of return to the 13-valent pneumococcal conjugate vaccine for adults in Turkey | TP | TN |
| Shami 2020 (116) | Evaluating the cost-effectiveness of a sequential pneumococcal vaccination compared to single-dose vaccination strategy for adults in Hong Kong | TP | TP |
| Shao 2020 (117) | Cost-effectiveness of pneumococcal vaccines among adults over 50 years old in low- and middle-income countries: a systematic review | TN | NA |
| Shiragami 2019 (118) | Cost-Effectiveness of the Adjuvant Recombinant Zoster Vaccine in Japanese Adults Aged 65 Years and Older | TN | NA |
| Shiri 2019 (119) | Pneumococcal Disease: A Systematic Review of Health Utilities, Resource Use, Costs, and Economic Evaluations of Interventions | TN | NA |
| Smith 2012 (120) | Cost-effectiveness of adult vaccination strategies using pneumococcal conjugate vaccine compared with pneumococcal polysaccharide vaccine | TP | TP |
| Smith 2013a (121) | Cost-effectiveness of pneumococcal conjugate vaccination in immunocompromised adults | TP | TP |
| Smith 2013b (122) | Modeling of cost effectiveness of pneumococcal conjugate vaccination strategies in U.S. older adults | TP | TP |
| Smith 2014 (123) | Determining the cost-effectiveness of adult pneumococcal vaccination strategies |  |  |
| Smith 2017 (124) | Cost-Effectiveness of the 4 Pillars Practice Transformation Program to Improve Vaccination of Adults Aged 65 and Older | FN | TP |
| Smith 2021a (125) | Higher-Valency Pneumococcal Conjugate Vaccines: An Exploratory Cost-Effectiveness Analysis in U.S. Seniors | TP | TP |
| Smith 2021b (126) | Should older adult pneumococcal vaccination recommendations change due to decreased vaccination in children during the pandemic? A cost-effectiveness analysis | TP | TN |
| Smith 2022 (127) | Cost-Effectiveness of Newly Recommended Pneumococcal Vaccination Strategies in Older Underserved Minority Adults in the USA | TP | TP |
| Stoecker 2016 (128) | Incremental Cost-Effectiveness of 13-valent Pneumococcal Conjugate Vaccine for Adults Age 50 Years and Older in the United States | FN | TP |
| Stoecker 2020 (129) | Cost-effectiveness of continuing pneumococcal conjugate vaccination at age 65 in the context of indirect effects from the childhood immunization program | TP | TP |
| Storch 2022 (130) | The effect of influenza and pneumococcal vaccination in the elderly on health service utilisation and costs: a claims data-based cohort study | TP | TN |
| Strutton 2012 (131) | Cost-effectiveness of 13-valent pneumococcal conjugate vaccine: Germany, Greece, and The Netherlands | TP | TN |
| Stuurman 2020 (132) | Vaccine effectiveness against laboratory-confirmed influenza in Europe - Results from the DRIVE network during season 2018/19 | TN | NA |
| Sun 2021 (133) | Cost-Effectiveness Analysis of 23-Valent Pneumococcal Polysaccharide Vaccine Program for the Elderly Aged 60 Years or Older in Shanghai, China | TP | TP |
| Thindwa 2021 (134) | Estimating the contribution of HIV-infected adults to household pneumococcal transmission in South Africa, 2016–2018: A hidden Markov modelling study | TN | NA |
| Thorrington 2018 (135) | Impact and cost-effectiveness of different vaccination strategies to reduce the burden of pneumococcal disease among elderly in the Netherlands | TP | TP |
| Tief 2016 (136) | An inception cohort study assessing the role of pneumococcal and other bacterial pathogens in children with influenza and ILI and a clinical decision model for stringent antibiotic use | TN | NA |
| Treskova 2019 (137) | Cost Effectiveness of Elderly Pneumococcal Vaccination in Presence of Higher-Valent Pneumococcal Conjugate Childhood Vaccination: Systematic Literature Review with Focus on Methods and Assumptions | TN | NA |
| Tyo 2011 (138) | Cost-effectiveness of conjugate pneumococcal vaccination in Singapore: comparing estimates for 7-valent, 10-valent, and 13-valent vaccines | FP | TN |
| van de Vooren 2014 (139) | Cost effectiveness of the new pneumococcal vaccines: a systematic review of European studies | TN | NA |
| van Hoek 2016 (140) | Cost-Effectiveness of Vaccinating Immunocompetent ≥65 Year Olds with the 13-Valent Pneumococcal Conjugate Vaccine in England | TP | TP |
| van Wekhoven (141) | The Community-Acquired Pneumonia immunization Trial in Adults (CAPiTA): what is the future of pneumococcal conjugate vaccination in elderly? | TN | NA |
| Vucina 2015 (142) | Cost-effectiveness of pneumococcal conjugate vaccination in Croatia | TN | NA |
| Wateska, 2018 (143) | Cost-effectiveness of increasing vaccination in high-risk adults aged 18-64 Years: a model-based decision analysis | FN | FN |
| Wateska 2019a (144) | An intervention to improve pneumococcal vaccination uptake in high risk 50-64 year olds vs. expanded age-based recommendations: an exploratory cost-effectiveness analysis | TP | TP |
| Wateska 2019b (145) | Cost-effectiveness of adult pneumococcal vaccination policies in underserved minorities aged 50-64 years compared to the US general population | TP | TP |
| Wateska 2020a (146) | Cost-Effectiveness of Pneumococcal Vaccination Policies and Uptake Programs in US Older Populations | TP | TP |
| Wateska 2020b (147) | Cost-Effectiveness of Pneumococcal Vaccination and Uptake Improvement Programs in Underserved and General Population Adults Aged < 65 Years | TP | FN |
| Wateska 2020c (148) | Pneumococcal Vaccination in Adults Aged ≥65 Years: Cost-Effectiveness and Health Impact in U.S. Populations | TP | TP |
| Wateska 2021 (149) | Is further research on adult pneumococcal vaccine uptake improvement programs worthwhile? Α value of information analysis | TP | FN |
| Wateska 2022 (150) | Cost-effectiveness of revised US pneumococcal vaccination recommendations in underserved minority adults < 65-years-old | TP | TP |
| Willem 2018 (151) | Economic evaluation of pneumococcal vaccines for adults aged over 50 years in Belgium | TP | TP |
| Wolff 2020 (152) | Cost-effectiveness of pneumococcal vaccination for elderly in Sweden | TP | TP |
| Wong 2017 (153) | Cost-effectiveness analysis of vaccinations and decision makings on vaccination programmes in Hong Kong: A systematic review | TN | NA |
| Woolfrey 2012 (154) | Pneumonia in Adults: The Practical Emergency Department Perspective | TN | NA |
| Wu 2013 (155) | Economic evaluation of universal 7-valent pneumococcal conjugate vaccination in Taiwan: a cost-effectiveness analysis | TP | TN |
| Xing 2019 (156) | Standing orders program of pneumococcal vaccination for hospitalized elderly patients in Hong Kong: A cost-effectiveness analysis | TP | TP |
| Zeevat 2019 (157) | Cost-effectiveness analysis on elderly pneumococcal vaccination in the Netherlands: Challenging the Dutch Health Council's advice | TP | FP |
| Zhao 2016 (158) | Cost-effectiveness of a 23-valent pneumococcal polysaccharide vaccine immunization programme for the elderly in Shanghai, China | TP | TP |
| Zhou 2018 (159) | Cost-effectiveness analysis of routine 13-valent pneumococcal conjugate vaccinations in Chinese infants | FP | TN |
| Zou 2022 (160) | Evaluation of effectiveness, safety and cost-benefit of the 23– valent pneumococcal capsular polysaccharide vaccine for HIV-Infected patients | FP | NA |

CDC, Centers for Disease Control and Prevention (United States); FN, false negative; FP, false positive; NA, not applicable; TN, true negative; TP, true positive.

^A^ ISLaR article inclusions and exclusions compared to human reviewer article inclusions and exclusions.

^B^ Five articles were missed by ISLaR when advancing studies included in the abstract screen to the full-text screen.

# References

1. Akazawa M, Yongue J, Ikeda S, Satoh T. Considering economic analyses in the revision of the preventive vaccination law: a new direction for health policy-making in Japan? Health Policy. 2014;118(1):127-34.

2. Akin L, Kaya M, Altinel S, Durand L. Cost of pneumococcal infections and cost-effectiveness analysis of pneumococcal vaccination at risk adults and elderly in Turkey. Hum Vaccin. 2011;7(4):441-50.

3. Aljunid S, Abuduxike G, Ahmed Z, Sulong S, Nur AM, Goh A. Impact of routine PCV7 (Prevenar) vaccination of infants on the clinical and economic burden of pneumococcal disease in Malaysia. BMC Infect Dis. 2011;11:248.

4. Ansaldi F, Pugh S, Amicizia D, Di Virgilio R, Trucchi C, Orsi A, et al. Estimating the Clinical and Economic Impact of Switching from the 13-Valent Pneumococcal Conjugate Vaccine (PCV13) to the 10-Valent Pneumococcal Conjugate Vaccine (PCV10) in Italy. Pathogens. 2020;9(2).

5. Astengo M, Paganino C, Amicizia D, Sticchi L, Orsi A, Icardi G, et al. Incidence of Hospitalisation and Emergency Department Visits for Pneumococcal Disease in Children, Adolescents, and Adults in Liguria, Italy: A Retrospective Analysis from 2012-2018. Vaccines (Basel). 2022;10(9).

6. Atwood M, Beausoleil L, Breton MC, Laferriere C, Sato R, Weycker D. Cost-effectiveness of alternative strategies for use of 13-valent pneumococcal conjugate vaccine (PCV13) in Canadian adults. Can J Public Health. 2018;109(5-6):756-68.

7. Bar-Zeev N, Kapanda L, King C, Beard J, Phiri T, Mvula H, et al. Methods and challenges in measuring the impact of national pneumococcal and rotavirus vaccine introduction on morbidity and mortality in Malawi. Vaccine. 2015;33(23):2637-45.

8. Berical AC, Harris D, Dela Cruz CS, Possick JD. Pneumococcal Vaccination Strategies. An Update and Perspective. Ann Am Thorac Soc. 2016;13(6):933-44.

9. Betsch C, Rossmann C, Pletz MW, Vollmar HC, Freytag A, Wichmann O, et al. Increasing influenza and pneumococcal vaccine uptake in the elderly: study protocol for the multi-methods prospective intervention study Vaccination60. BMC Public Health. 2018;18(1):885.

10. Biagini L, Pezzani M, Rojas R, Fuentealba F. Cost-Utility Study of PCV13 Versus PPSV23 in Adults in Chile. Value Health Reg Issues. 2018;17:194-201.

11. Birck AM, Nordin Christensen L, Pedersen MH, Olsen J, Johnson KD, Bencina G, et al. Health economic evaluation of introducing a PPSV23-based vaccination programme to adults aged 65 and above, and an extension to the 60-64 age group in Denmark. Expert Rev Vaccines. 2021;20(10):1327-37.

12. Blank PR, Szucs TD. Cost-effectiveness of 13-valent pneumococcal conjugate vaccine in Switzerland. Vaccine. 2012;30(28):4267-75.

13. Blasi F, Mantero M, Santus P, Tarsia P. Understanding the burden of pneumococcal disease in adults. Clin Microbiol Infect. 2012;18 Suppl 5:7-14.

14. Blommaert A, Bilcke J, Willem L, Verhaegen J, Goossens H, Beutels P. The cost-effectiveness of pneumococcal vaccination in healthy adults over 50: An exploration of influential factors for Belgium. Vaccine. 2016;34(18):2106-12.

15. Boccalini S, Bechini A, Levi M, Tiscione E, Gasparini R, Bonanni P. Cost-effectiveness of new adult pneumococcal vaccination strategies in Italy. Hum Vaccin Immunother. 2013;9(3):699-706.

16. Boccalini S, Bechini A, Gasparini R, Panatto D, Amicizia D, Bonanni P. Economic studies applied to vaccines against invasive diseases: An updated budget impact analysis of age-based pneumococcal vaccination strategies in the elderly in Italy. Hum Vaccin Immunother. 2017;13(2):417-22.

17. Bolaños-Díaz R, Miño-León G, Zea E. Cost-effectiveness of the 13-valent pneumococcal conjugate vaccine compared to the 10-valent vaccine in children: predictive analysis in the Ecuadorian context. Journal of Pharmaceutical Health Services Research. 2022;13(4):341-50.

18. Buchwald UK, Folaranmi T, Weiss T. Response to Shami et al. 'Evaluating the cost-effectiveness of a sequential pneumococcal vaccination compared to single dose vaccination strategy for adults in Hong Kong' (Hum Vacc Immunother 2020). Hum Vaccin Immunother. 2021;17(1):173-5.

19. Caldwell R, Roberts CS, An Z, Chen CI, Wang B. The health and economic impact of vaccination with 7-valent pneumococcal vaccine (PCV7) during an annual influenza epidemic and influenza pandemic in China. BMC Infect Dis. 2015;15:284.

20. Castaneda-Orjuela C, Alvis-Guzman N, Paternina AJ, De la Hoz-Restrepo F. Cost-effectiveness of the introduction of the pneumococcal polysaccharide vaccine in elderly Colombian population. Vaccine. 2011;29(44):7644-50.

21. Chaiyakunapruk N, Song D, Lynch J, Kim JH, Dilokthornsakul P, Chotpitayasunondh T, et al. Public Health Value of a Hypothetical Pneumococcal Conjugate Vaccine (PCV) Introduction: A Case Study. Vaccines (Basel). 2022;10(6).

22. Centers for Disease Control and Prevention. Use of 13-valent pneumococcal conjugate vaccine and 23-valent pneumococcal polysaccharide vaccine for adults with immunocompromising conditions: recommendations of the Advisory Committee on Immunization Practices (ACIP). MMWR Morb Mortal Wkly Rep. 2012;61(40):816-9.

23. Chen J, O'Brien MA, Yang HK, Grabenstein JD, Dasbach EJ. Cost-effectiveness of pneumococcal vaccines for adults in the United States. Adv Ther. 2014;31(4):392-409.

24. Chen C, Beutels P, Newall AT. Evolution over time in the cost-effectiveness of pneumococcal conjugate vaccine (PCV13) in older Australians due to herd protection from infant vaccination. Vaccine. 2018;36(16):2057-60.

25. Chen C, Beutels P, Wood J, Menzies R, MacIntyre CR, McIntyre P, et al. Retrospective cost-effectiveness of the 23-valent pneumococcal polysaccharide vaccination program in Australia. Vaccine. 2018;36(42):6307-13.

26. Chen C, Wood JG, Beutels P, Menzies R, MacIntyre CR, Dirmesropian S, et al. The role of timeliness in the cost-effectiveness of older adult vaccination: A case study of pneumococcal conjugate vaccine in Australia. Vaccine. 2018;36(10):1265-71.

27. Chen D, Ye Z, Pi Z, Mizukami S, Aoyagi K, Jiang Y. Cost-effectiveness of dual influenza and pneumococcal vaccination among the elderly in Shenzhen, China. Vaccine. 2021;39(16):2237-45.

28. Cho BH, Stoecker C, Link-Gelles R, Moore MR. Cost-effectiveness of administering 13-valent pneumococcal conjugate vaccine in addition to 23-valent pneumococcal polysaccharide vaccine to adults with immunocompromising conditions. Vaccine. 2013;31(50):6011-21.

29. Choi MJ, Kang SO, Oh JJ, Park SB, Kim MJ, Cheong HJ. Cost-effectiveness analysis of 13-valent pneumococcal conjugate vaccine versus 23-valent pneumococcal polysaccharide vaccine in an adult population in South Korea. Hum Vaccin Immunother. 2018;14(8):1914-22.

30. Christie D, Viner RM, Knox K, Coen PG, Wang H, El Bashir H, et al. Long-term outcomes of pneumococcal meningitis in childhood and adolescence. Eur J Pediatr. 2011;170(8):997-1006.

31. Dao MD, Nguyen ST, Ha TV, Pham LD, Vo TQ, Trung Q. Socioeconomic Burden of Community-acquired Pneumonia Associated Hospitalizations among Vietnamese Patients: A Prospective, Incidence-based Study. Asian Journal of Pharmaceutics. 2018;12(1):S38-S47.

32. Deb A, Guggisberg P, Mutschler T, Owusu-Edusei K, Bencina G, Johnson KD, et al. Cost-effectiveness of the 15-valent pneumococcal conjugate vaccine for high-risk adults in Switzerland. Expert Rev Vaccines. 2022;21(5):711-22.

33. de Soarez PC, Sartori AM, Freitas AC, Nishikawa AM, Novaes HM. Cost-Effectiveness Analysis of Universal Vaccination of Adults Aged 60 Years with 23-Valent Pneumococcal Polysaccharide Vaccine versus Current Practice in Brazil. PLoS One. 2015;10(6):e0130217.

34. de Vries LM, Kellerborg KM, Brouwer WBF, van Baal PHM. Don't forget about the future: The impact of including future costs on the cost-effectiveness of adult pneumococcal conjugate vaccination with PCV13 in the Netherlands. Vaccine. 2021;39(29):3834-43.

35. Diez-Domingo J, Ridao-Lopez M, Gutierrez-Gimeno MV, Puig-Barbera J, Lluch-Rodrigo JA, Pastor-Villalba E. Pharmacoeconomic assessment of implementing a universal PCV-13 vaccination programme in the Valencian public health system (Spain). Vaccine. 2011;29(52):9640-8.

36. Dilokthornsakul P, Kengkla K, Saokaew S, Permsuwan U, Techasaensiri C, Chotpitayasunondh T, et al. An updated cost-effectiveness analysis of pneumococcal conjugate vaccine among children in Thailand. Vaccine. 2019;37(32):4551-60.

37. Ding H, Huang J, Ngai CH, Sun Q, Kwok KO, Wang HH, et al. The cost-effectiveness of starting 23-valent pneumococcal polysaccharide vaccine and influenza vaccination at 50 vs. 65 years: A comparative modelling study. Vaccine. 2022;40(9):1282-8.

38. Dirmesropian S, Wood JG, MacIntyre CR, Newall AT. A review of economic evaluations of 13-valent pneumococcal conjugate vaccine (PCV13) in adults and the elderly. Hum Vaccin Immunother. 2015;11(4):818-25.

39. Dirmesropian S, Wood JG, MacIntyre CR, Beutels P, McIntyre P, Menzies R, et al. Cost-effectiveness of 13-valent pneumococcal conjugate vaccine (PCV13) in older Australians. Vaccine. 2017;35(34):4307-14.

40. Dominguez A, Castilla J, Godoy P, Delgado-Rodriguez M, Saez M, Soldevila N, et al. Effectiveness of vaccination with 23-valent pneumococcal polysaccharide vaccine in preventing hospitalization with laboratory confirmed influenza during the 2009-2010 and 2010-2011 seasons. Hum Vaccin Immunother. 2013;9(4):865-73.

41. Dorji K, Phuntsho S, Pempa, Kumluang S, Khuntha S, Kulpeng W, et al. Towards the introduction of pneumococcal conjugate vaccines in Bhutan: A cost-utility analysis to determine the optimal policy option. Vaccine. 2018;36(13):1757-65.

42. Earnshaw SR, McDade CL, Zanotti G, Farkouh RA, Strutton D. Cost-effectiveness of 2 + 1 dosing of 13-valent and 10-valent pneumococcal conjugate vaccines in Canada. BMC Infect Dis. 2012;12:101.

43. Eilers R, Krabbe PF, van Essen TG, Suijkerbuijk A, van Lier A, de Melker HE. Assessment of vaccine candidates for persons aged 50 and older: a review. BMC Geriatr. 2013;13:32.

44. Eythorsson E, Asgeirsdottir TL, Erlendsdottir H, Hrafnkelsson B, Kristinsson KG, Haraldsson A. The impact and cost-effectiveness of introducing the 10-valent pneumococcal conjugate vaccine into the paediatric immunisation programme in Iceland-A population-based time series analysis. PLoS One. 2021;16(4):e0249497.

45. Feemster KA, Kim Y, Abe M, Johnson K, Sasaki S. Response to Igarashi, et al, cost-effectiveness analysis for PCV13 in adults 60 years and over with underlying medical conditions which put them at an elevated risk of pneumococcal disease in Japan. Expert Rev Vaccines. 2022;21(5):589-90.

46. Feldman C, Dlamini SK, Madhi SA, Meiring S, von Gottberg A, de Beer JC, et al. The cost-effectiveness of using pneumococcal conjugate vaccine (PCV13) versus pneumococcal polysaccharide vaccine (PPSV23), in South African adults. PLoS One. 2020;15(1):e0227945.

47. Gaillat J. Compared effectiveness of the 7-valent pneumococcal conjugate vaccine in children with the 13-valent vaccine in adults. Med Mal Infect. 2013;43(6):215-21.

48. Gamil A, Chokephaibulkit K, Phongsamart W, Techasaensiri C, Piralam B, Thamaree R. Pneumococcal disease in Thailand. Int J Infect Dis. 2021;102:429-36.

49. Giglio ND, Castellano VE, Mizrahi P, Micone PV. Cost-Effectiveness of Pneumococcal Vaccines for Adults Aged 65 Years and Older in Argentina. Value Health Reg Issues. 2022;28:76-81.

50. Gil de Miguel A, Eiros Bouza JM, Martinez Alcorta LI, Callejo D, Minarro C, Vallejo-Aparicio LA, et al. Direct Medical Costs of Four Vaccine-Preventable Infectious Diseases in Older Adults in Spain. Pharmacoecon Open. 2022;6(4):509-18.

51. Gilchrist SA, Nanni A, Levine O. Benefits and effectiveness of administering pneumococcal polysaccharide vaccine with seasonal influenza vaccine: an approach for policymakers. Am J Public Health. 2012;102(4):596-605.

52. Gouveia M, Fiorentino F, Jesus G, Costa J, Borges M. Cost-effectiveness of the 13-valent Pneumococcal Conjugate Vaccine in Children in Portugal. Pediatr Infect Dis J. 2017;36(8):782-7.

53. Gouveia M, Jesus G, Ines M, Costa J, Borges M. Cost-effectiveness of the 13-valent pneumococcal conjugate vaccine in adults in Portugal versus "no vaccination" and versus vaccination with the 23-valent pneumococcal polysaccharide vaccine. Hum Vaccin Immunother. 2019;15(4):850-8.

54. Grzesiowski P, Aguiar-Ibanez R, Kobryn A, Durand L, Puig PE. Cost-effectiveness of polysaccharide pneumococcal vaccination in people aged 65 and above in Poland. Hum Vaccin Immunother. 2012;8(10):1382-94.

55. Haasis MA, Ceria JA, Kulpeng W, Teerawattananon Y, Alejandria M. Do Pneumococcal Conjugate Vaccines Represent Good Value for Money in a Lower-Middle Income Country? A Cost-Utility Analysis in the Philippines. PLoS One. 2015;10(7):e0131156.

56. Hall VJ, Foulkes S, Saei A, Andrews N, Oguti B, Charlett A, et al. COVID-19 vaccine coverage in health-care workers in England and effectiveness of BNT162b2 mRNA vaccine against infection (SIREN): a prospective, multicentre, cohort study. Lancet. 2021;397(10286):1725-35.

57. Heo JY, Seo YB, Choi WS, Lee J, Noh JY, Jeong HW, et al. Cost-effectiveness of pneumococcal vaccination strategies for the elderly in Korea. PLoS One. 2017;12(5):e0177342.

58. Hoshi SL, Kondo M, Okubo I. Economic Evaluation of Immunisation Programme of 23-Valent Pneumococcal Polysaccharide Vaccine and the Inclusion of 13-Valent Pneumococcal Conjugate Vaccine in the List for Single-Dose Subsidy to the Elderly in Japan. PLoS One. 2015;10(10):e0139140.

59. Hoshi SL, Shono A, Seposo X, Okubo R, Kondo M. Cost-effectiveness analyses of 15- and 20-valent pneumococcal conjugate vaccines for Japanese elderly. Vaccine. 2022;40(49):7057-64.

60. Igarashi A, Hirose E, Kobayashi Y, Yonemoto N, Lee B. Cost-effectiveness analysis for PCV13 in adults 60 years and over with underlying medical conditions which put them at an elevated risk of pneumococcal disease in Japan. Expert Rev Vaccines. 2021;20(9):1153-65.

61. Igarashi A, Hirose E, Kobayashi Y, Yonemoto N, Lee B. Response to Feemster KA et al. letter to the editor on Cost-effectiveness analysis for PCV13 in adults 60 years and over with underlying medical conditions which put them at an elevated risk of pneumococcal disease in Japan. Expert Rev Vaccines. 2022;21(5):591-2.

62. Ignatova GL, Avdeev SN, Antonov VN. Comparative effectiveness of pneumococcal vaccination with PPV23 and PCV13 in COPD patients over a 5-year follow-up cohort study. Sci Rep. 2021;11(1):15948.

63. Ishigami J, Padula WV, Grams ME, Chang AR, Jaar B, Gansevoort RT, et al. Cost-effectiveness of Pneumococcal Vaccination Among Patients With CKD in the United States. Am J Kidney Dis. 2019;74(1):23-35.

64. Jiang Y, Gauthier A, Annemans L, van der Linden M, Nicolas-Spony L, Bresse X. Cost-effectiveness of vaccinating adults with the 23-valent pneumococcal polysaccharide vaccine (PPV23) in Germany. Expert Rev Pharmacoecon Outcomes Res. 2012;12(5):645-60.

65. Jiang Y, Gauthier A, Keeping S, Carroll S. Cost-effectiveness of vaccinating the elderly and at-risk adults with the 23-valent pneumococcal polysaccharide vaccine or 13-valent pneumococcal conjugate vaccine in the UK. Expert Rev Pharmacoecon Outcomes Res. 2014;14(6):913-27.

66. Jiang Y, Yang X, Taniguchi K, Petigara T, Abe M. A cost-effectiveness analysis of revaccination and catch-up strategies with the 23-valent pneumococcal polysaccharide vaccine (PPV23) in older adults in Japan. J Med Econ. 2018;21(7):687-97.

67. Jose RJ, Brown JS. Adult pneumococcal vaccination: advances, impact, and unmet needs. Curr Opin Pulm Med. 2017;23(3):225-30.

68. Klok RM, Lindkvist RM, Ekelund M, Farkouh RA, Strutton DR. Cost-effectiveness of a 10- versus 13-valent pneumococcal conjugate vaccine in Denmark and Sweden. Clin Ther. 2013;35(2):119-34.

69. Knerer G, Ismaila A, Pearce D. Health and economic impact of PHiD-CV in Canada and the UK: a Markov modelling exercise. J Med Econ. 2012;15(1):61-76.

70. Kobayashi M, Stoecker C, Xing W, Cho BH, Pilishvili T. Cost-effectiveness of implementing 13-valent pneumococcal conjugate vaccine for U.S. adults aged 19 years and older with underlying conditions. Hum Vaccin Immunother. 2021;17(7):2232-40.

71. Kuchenbecker U, Chase D, Reichert A, Schiffner-Rohe J, Atwood M. Estimating the cost-effectiveness of a sequential pneumococcal vaccination program for adults in Germany. PLoS One. 2018;13(5):e0197905.

72. Kuhlmann A, von der Schulenburg JG. Modeling the cost-effectiveness of infant vaccination with pneumococcal conjugate vaccines in Germany. Eur J Health Econ. 2017;18(3):273-92.

73. Kulpeng W, Leelahavarong P, Rattanavipapong W, Sornsrivichai V, Baggett HC, Meeyai A, et al. Cost-utility analysis of 10- and 13-valent pneumococcal conjugate vaccines: protection at what price in the Thai context? Vaccine. 2013;31(26):2839-47.

74. Lawrence H, Pick H, Baskaran V, Daniel P, Rodrigo C, Ashton D, et al. Effectiveness of the 23-valent pneumococcal polysaccharide vaccine against vaccine serotype pneumococcal pneumonia in adults: A case-control test-negative design study. PLoS Med. 2020;17(10):e1003326.

75. Leidner AJ, Murthy N, Chesson HW, Biggerstaff M, Stoecker C, Harris AM, et al. Cost-effectiveness of adult vaccinations: A systematic review. Vaccine. 2019;37(2):226-34.

76. Liguori G, Parlato A, Zamparelli AS, Belfiore P, Galle F, Di Onofrio V, et al. Adult immunization with 13-valent pneumococcal vaccine in Campania region, South Italy: an economic evaluation. Hum Vaccin Immunother. 2014;10(2):492-7.

77. Lin CJ, Zimmerman RK, Smith KJ. Cost-effectiveness of pneumococcal and influenza vaccination standing order programs. Am J Manag Care. 2013;19(1):e30-7.

78. Mangen MJ, Rozenbaum MH, Huijts SM, van Werkhoven CH, Postma DF, Atwood M, et al. Cost-effectiveness of adult pneumococcal conjugate vaccination in the Netherlands. Eur Respir J. 2015;46(5):1407-16.

79. Marbaix S, Peetermans WE, Verhaegen J, Annemans L, Sato R, Mignon A, et al. Cost-effectiveness of PCV13 vaccination in Belgian adults aged 65-84 years at elevated risk of pneumococcal infection. PLoS One. 2018;13(7):e0199427.

80. Mauskopf J, Blake L, Eiden A, Roberts C, Hu T, Nyaku M. Economic Evaluation of Vaccination Programs: A Guide for Selecting Modeling Approaches. Value Health. 2022;25(5):810-23.

81. McGarry LJ, Gilmore KE, Rubin JL, Klugman KP, Strutton DR, Weinstein MC. Impact of 13-valent pneumococcal conjugate vaccine (PCV13) in a pandemic similar to the 2009 H1N1 in the United States. BMC Infect Dis. 2013;13:229.

82. Mendes D, Averin A, Atwood M, Sato R, Vyse A, Campling J, et al. Cost-effectiveness of using a 20-valent pneumococcal conjugate vaccine to directly protect adults in England at elevated risk of pneumococcal disease. Expert Rev Pharmacoecon Outcomes Res. 2022;22(8):1285-95.

83. Michaelidis CI, Zimmerman RK, Nowalk MP, Smith KJ. Cost-effectiveness of a program to eliminate disparities in pneumococcal vaccination rates in elderly minority populations: an exploratory analysis. Value Health. 2013;16(2):311-7.

84. Migdal VL, Harper K, Haqqani N, Janiak B. Time Cost of Standardized Nursing Screens in the Emergency Department. West J Emerg Med. 2019;20(6):851-4.

85. Musher DM, Rodriguez-Barradas MB. Why the recent ACIP recommendations regarding conjugate pneumococcal vaccine in adults may be irrelevant. Hum Vaccin Immunother. 2016;12(2):331-5.

86. Nakamura MM, Tasslimi A, Lieu TA, Levine O, Knoll MD, Russell LB, et al. Cost effectiveness of child pneumococcal conjugate vaccination in middle-income countries. Int Health. 2011;3(4):270-81.

87. Namkoong H, Ishii M, Funatsu Y, Kimizuka Y, Yagi K, Asami T, et al. Theory and strategy for Pneumococcal vaccines in the elderly. Hum Vaccin Immunother. 2016;12(2):336-43.

88. Naucler P, Galanis I, Morfeldt E, Darenberg J, Ortqvist A, Henriques-Normark B. Comparison of the Impact of Pneumococcal Conjugate Vaccine 10 or Pneumococcal Conjugate Vaccine 13 on Invasive Pneumococcal Disease in Equivalent Populations. Clin Infect Dis. 2017;65(11):1780-9.

89. Neto JT, de Araujo GT, Gagliardi A, Pinho A, Durand L, Fonseca M. Cost-effectiveness analysis of pneumococcal polysaccharide vaccination from age 60 in Sao Paulo State, Brazil. Hum Vaccin. 2011;7(10):1037-47.

90. Newall AT, Reyes JF, McIntyre P, Menzies R, Beutels P, Wood JG. Retrospective economic evaluation of childhood 7-valent pneumococcal conjugate vaccination in Australia: Uncertain herd impact on pneumonia critical. Vaccine. 2016;34(3):320-7.

91. Newall AT. What do we know about the cost-effectiveness of pneumococcal conjugate vaccination in older adults? Hum Vaccin Immunother. 2016;12(10):2666-9.

92. Newall AT, Wood JG, Chen C, McIntyre P, Beutels P. Reply to letter: Retrospective cost-effectiveness of the 23-valent pneumococcal polysaccharide vaccination program in Australia. Vaccine. 2019;37(52):7534.

93. Nicholson KG, Abrams KR, Batham S, Medina MJ, Warren FC, Barer M, et al. Randomised controlled trial and health economic evaluation of the impact of diagnostic testing for influenza, respiratory syncytial virus and Streptococcus pneumoniae infection on the management of acute admissions in the elderly and high-risk 18- to 64-year-olds. Health Technol Assess. 2014;18(36):1-274, vii-viii.

94. Nishikawa AM, Sartori AMC, Mainardi GM, Freitas AC, Itria A, Novaes HMD, et al. Systematic review of economic evaluations of the 23-valent pneumococcal polysaccharide vaccine (PPV23) in individuals 60 years of age or older. Vaccine. 2018;36(19):2510-22.

95. Nymark LS, Dag Berild J, Lyngstad TM, Askeland Winje B, Frimann Vestrheim D, Aaberge I, et al. Cost-utility analysis of the universal pneumococcal vaccination programme for older adults in Norway. Hum Vaccin Immunother. 2022;18(6):2101333.

96. Olsen J, Schnack H, Skovdal M, Vietri J, Mikkelsen MB, Poulsen PB. Cost-effectiveness of 20-valent pneumococcal conjugate vaccine in Denmark compared with PPV23. J Med Econ. 2022;25(1):1240-54.

97. Ordonez JE, Orozco JJ. Cost-effectiveness analysis of pneumococcal conjugate vaccine 13-valent in older adults in Colombia. BMC Infect Dis. 2014;14:172.

98. Perdrizet J, Santana CFS, Senna T, Alexandre RF, Sini de Almeida R, Spinardi J, et al. Cost-effectiveness analysis of replacing the 10-valent pneumococcal conjugate vaccine (PCV10) with the 13-valent pneumococcal conjugate vaccine (PCV13) in Brazil infants. Hum Vaccin Immunother. 2021;17(4):1162-72.

99. Piralam B, Tomczyk SM, Rhodes JC, Thamthitiwat S, Gregory CJ, Olsen SJ, et al. Incidence of Pneumococcal Pneumonia Among Adults in Rural Thailand, 2006-2011: Implications for Pneumococcal Vaccine Considerations. Am J Trop Med Hyg. 2015;93(6):1140-7.

100. Pircon JY, Talarico CA, Bollaerts K, Hausdorff WP, Clarke CJ. The choice of analytical methodology can alter conclusions regarding herd effects of paediatric pneumococcal vaccination programmes. Vaccine. 2018;36(46):6933-43.

101. Pitsiou GG, Kioumis IP. Pneumococcal vaccination in adults: does it really work? Respir Med. 2011;105(12):1776-83.

102. Pletz MW, Welte T. Pneumococcal conjugate vaccine for adults: "It's tough to make predictions, ...". Eur Respir J. 2015;46(5):1265-8.

103. Plosker GL. 13-Valent Pneumococcal Conjugate Vaccine: A Review of Its Use in Adults. Drugs. 2015;75(13):1535-46.

104. Polistena B, Icardi G, Orsi A, Spandonaro F, Di Virgilio R, d'Angela D. Cost-Effectiveness of Vaccination with the 20-Valent Pneumococcal Conjugate Vaccine in the Italian Adult Population. Vaccines (Basel). 2022;10(12).

105. Porchia BR, Bonanni P, Bechini A, Bonaccorsi G, Boccalini S. Evaluating the costs and benefits of pneumococcal vaccination in adults. Expert Rev Vaccines. 2017;16(2):93-107.

106. Pradas R, Gil de Miguel A, Alvaro A, Gil-Prieto R, Lorente R, Mendez C, et al. Budget impact analysis of a pneumococcal vaccination programme in the 65-year-old Spanish cohort using a dynamic model. BMC Infect Dis. 2013;13:175.

107. Pugh S, Wasserman M, Moffatt M, Marques S, Reyes JM, Prieto VA, et al. Estimating the Impact of Switching from a Lower to Higher Valent Pneumococcal Conjugate Vaccine in Colombia, Finland, and The Netherlands: A Cost-Effectiveness Analysis. Infect Dis Ther. 2020;9(2):305-24.

108. Ren S, Attia J, Li SC, Newby D. Pneumococcal polysaccharide vaccine is a cost saving strategy for prevention of acute coronary syndrome. Vaccine. 2021;39(12):1721-6.

109. Robberstad B, Frostad CR, Akselsen PE, Kvaerner KJ, Berstad AK. Economic evaluation of second generation pneumococcal conjugate vaccines in Norway. Vaccine. 2011;29(47):8564-74.

110. Rodgers GL, Klugman KP. Surveillance of the impact of pneumococcal conjugate vaccines in developing countries. Hum Vaccin Immunother. 2016;12(2):417-20.

111. Rodriguez Gonzalez-Moro JM, Menendez R, Campins M, Lwoff N, Oyaguez I, Echave M, et al. Cost Effectiveness of the 13-Valent Pneumococcal Conjugate Vaccination Program in Chronic Obstructive Pulmonary Disease Patients Aged 50+ Years in Spain. Clin Drug Investig. 2016;36(1):41-53.

112. Rozenbaum MH, van Hoek AJ, Fleming D, Trotter CL, Miller E, Edmunds WJ. Vaccination of risk groups in England using the 13 valent pneumococcal conjugate vaccine: economic analysis. BMJ. 2012;345:e6879.

113. Sanduzzi A, Canora A, Belfiore P, Bocchino M, Liguori R, Liguori G. Impact of 13Valent Vaccine for Prevention of Pneumococcal Diseases in Children and Adults at Risk: Possible Scenarios in Campania Region. Infect Disord Drug Targets. 2019;19(4):403-8.

114. Sehatzadeh S. Influenza and pneumococcal vaccinations for patients with chronic obstructive pulmonary disease (COPD): an evidence-based review. Ont Health Technol Assess Ser. 2012;12(3):1-64.

115. Sevilla JP, Stawasz A, Burnes D, Agarwal A, Hacibedel B, Helvacioglu K, et al. Indirect costs of adult pneumococcal disease and the productivity-based rate of return to the 13-valent pneumococcal conjugate vaccine for adults in Turkey. Hum Vaccin Immunother. 2020;16(8):1923-36.

116. Shami JJP, Pathadka S, Chan EW, Hui J, Sato R, Patil S, et al. Evaluating the cost-effectiveness of a sequential pneumococcal vaccination compared to single-dose vaccination strategy for adults in Hong Kong. Hum Vaccin Immunother. 2020;16(8):1937-44.

117. Shao Y, Stoecker C. Cost-effectiveness of pneumococcal vaccines among adults over 50 years old in low- and middle-income countries: a systematic review. Expert Rev Vaccines. 2020;19(12):1141-51.

118. Shiragami M, Mizukami A, Kaise T, Curran D, Van Oorschot D, Bracke B, et al. Cost-Effectiveness of the Adjuvant Recombinant Zoster Vaccine in Japanese Adults Aged 65 Years and Older. Dermatol Ther (Heidelb). 2019;9(2):281-97.

119. Shiri T, Khan K, Keaney K, Mukherjee G, McCarthy ND, Petrou S. Pneumococcal Disease: A Systematic Review of Health Utilities, Resource Use, Costs, and Economic Evaluations of Interventions. Value Health. 2019;22(11):1329-44.

120. Smith KJ, Wateska AR, Nowalk MP, Raymund M, Nuorti JP, Zimmerman RK. Cost-effectiveness of adult vaccination strategies using pneumococcal conjugate vaccine compared with pneumococcal polysaccharide vaccine. JAMA. 2012;307(8):804-12.

121. Smith KJ, Nowalk MP, Raymund M, Zimmerman RK. Cost-effectiveness of pneumococcal conjugate vaccination in immunocompromised adults. Vaccine. 2013;31(37):3950-6.

122. Smith KJ, Wateska AR, Nowalk MP, Raymund M, Lee BY, Zimmerman RK. Modeling of cost effectiveness of pneumococcal conjugate vaccination strategies in U.S. older adults. Am J Prev Med. 2013;44(4):373-81.

123. Smith KJ. Determining the cost-effectiveness of adult pneumococcal vaccination strategies. Expert Rev Pharmacoecon Outcomes Res. 2014;14(1):1-4.

124. Smith KJ, Zimmerman RK, Nowalk MP, Lin CJ. Cost-Effectiveness of the 4 Pillars Practice Transformation Program to Improve Vaccination of Adults Aged 65 and Older. J Am Geriatr Soc. 2017;65(4):763-8.

125. Smith KJ, Wateska AR, Nowalk MP, Lin CJ, Harrison LH, Schaffner W, et al. Higher-Valency Pneumococcal Conjugate Vaccines: An Exploratory Cost-Effectiveness Analysis in U.S. Seniors. Am J Prev Med. 2021;61(1):28-36.

126. Smith KJ, Wateska AR, Nowalk MP, Lin CJ, Harrison LH, Schaffner W, et al. Should older adult pneumococcal vaccination recommendations change due to decreased vaccination in children during the pandemic? A cost-effectiveness analysis. Vaccine. 2021;39(31):4278-82.

127. Smith KJ, Wateska AR, Nowalk MP, Lin CJ, Harrison LH, Schaffner W, et al. Cost-Effectiveness of Newly Recommended Pneumococcal Vaccination Strategies in Older Underserved Minority Adults in the USA. Infect Dis Ther. 2022;11(4):1683-93.

128. Stoecker C, Kim L, Gierke R, Pilishvili T. Incremental Cost-Effectiveness of 13-valent Pneumococcal Conjugate Vaccine for Adults Age 50 Years and Older in the United States. J Gen Intern Med. 2016;31(8):901-8.

129. Stoecker C, Kobayashi M, Matanock A, Cho BH, Pilishvili T. Cost-effectiveness of continuing pneumococcal conjugate vaccination at age 65 in the context of indirect effects from the childhood immunization program. Vaccine. 2020;38(7):1770-7.

130. Storch J, Fleischmann-Struzek C, Rose N, Lehmann T, Mikolajetz A, Maddela S, et al. The effect of influenza and pneumococcal vaccination in the elderly on health service utilisation and costs: a claims data-based cohort study. Eur J Health Econ. 2022;23(1):67-80.

131. Strutton DR, Farkouh RA, Earnshaw SR, Hwang S, Theidel U, Kontodimas S, et al. Cost-effectiveness of 13-valent pneumococcal conjugate vaccine: Germany, Greece, and The Netherlands. J Infect. 2012;64(1):54-67.

132. Stuurman AL, Bollaerts K, Alexandridou M, Biccler J, Diez Domingo J, Nohynek H, et al. Vaccine effectiveness against laboratory-confirmed influenza in Europe - Results from the DRIVE network during season 2018/19. Vaccine. 2020;38(41):6455-63.

133. Sun X, Tang Y, Ma X, Guo X, Huang Z, Ren J, et al. Cost-Effectiveness Analysis of 23-Valent Pneumococcal Polysaccharide Vaccine Program for the Elderly Aged 60 Years or Older in Shanghai, China. Front Public Health. 2021;9:647725.

134. Thindwa D, Wolter N, Pinsent A, Carrim M, Ojal J, Tempia S, et al. Estimating the contribution of HIV-infected adults to household pneumococcal transmission in South Africa, 2016-2018: A hidden Markov modelling study. PLoS Comput Biol. 2021;17(12):e1009680.

135. Thorrington D, van Rossum L, Knol M, de Melker H, Rumke H, Hak E, et al. Impact and cost-effectiveness of different vaccination strategies to reduce the burden of pneumococcal disease among elderly in the Netherlands. PLoS One. 2018;13(2):e0192640.

136. Tief F, Hoppe C, Seeber L, Obermeier P, Chen X, Karsch K, et al. An inception cohort study assessing the role of pneumococcal and other bacterial pathogens in children with influenza and ILI and a clinical decision model for stringent antibiotic use. Antivir Ther. 2016;21(5):413-24.

137. Treskova M, Scholz SM, Kuhlmann A. Cost Effectiveness of Elderly Pneumococcal Vaccination in Presence of Higher-Valent Pneumococcal Conjugate Childhood Vaccination: Systematic Literature Review with Focus on Methods and Assumptions. Pharmacoeconomics. 2019;37(9):1093-127.

138. Tyo KR, Rosen MM, Zeng W, Yap M, Pwee KH, Ang LW, et al. Cost-effectiveness of conjugate pneumococcal vaccination in Singapore: comparing estimates for 7-valent, 10-valent, and 13-valent vaccines. Vaccine. 2011;29(38):6686-94.

139. van de Vooren K, Duranti S, Curto A, Garattini L. Cost effectiveness of the new pneumococcal vaccines: a systematic review of European studies. Pharmacoeconomics. 2014;32(1):29-45.

140. van Hoek AJ, Miller E. Cost-Effectiveness of Vaccinating Immunocompetent >/=65 Year Olds with the 13-Valent Pneumococcal Conjugate Vaccine in England. PLoS One. 2016;11(2):e0149540.

141. van Werkhoven CH, Bonten MJ. The Community-Acquired Pneumonia immunization Trial in Adults (CAPiTA): what is the future of pneumococcal conjugate vaccination in elderly? Future Microbiol. 2015;10(9):1405-13.

142. Vucina VV, Filipovic SK, Koznjak N, Stamenic V, Clark AD, Mounaud B, et al. Cost-effectiveness of pneumococcal conjugate vaccination in Croatia. Vaccine. 2015;33 Suppl 1:A209-18.

143. Wateska AR, Nowalk MP, Zimmerman RK, Smith KJ, Lin CJ. Cost-effectiveness of increasing vaccination in high-risk adults aged 18-64 Years: a model-based decision analysis. BMC Infect Dis. 2018;18(1):52.

144. Wateska AR, Nowalk MP, Lin CJ, Harrison LH, Schaffner W, Zimmerman RK, et al. An intervention to improve pneumococcal vaccination uptake in high risk 50-64 year olds vs. expanded age-based recommendations: an exploratory cost-effectiveness analysis. Hum Vaccin Immunother. 2019;15(4):863-72.

145. Wateska AR, Nowalk MP, Lin CJ, Harrison LH, Schaffner W, Zimmerman RK, et al. Cost-effectiveness of adult pneumococcal vaccination policies in underserved minorities aged 50-64 years compared to the US general population. Vaccine. 2019;37(14):2026-33.

146. Wateska AR, Nowalk MP, Lin CJ, Harrison LH, Schaffner W, Zimmerman RK, et al. Cost-Effectiveness of Pneumococcal Vaccination Policies and Uptake Programs in US Older Populations. J Am Geriatr Soc. 2020;68(6):1271-8.

147. Wateska AR, Nowalk MP, Lin CJ, Harrison LH, Schaffner W, Zimmerman RK, et al. Cost-Effectiveness of Pneumococcal Vaccination and Uptake Improvement Programs in Underserved and General Population Adults Aged < 65 Years. J Community Health. 2020;45(1):111-20.

148. Wateska AR, Nowalk MP, Lin CJ, Harrison LH, Schaffner W, Zimmerman RK, et al. Pneumococcal Vaccination in Adults Aged >/=65 Years: Cost-Effectiveness and Health Impact in U.S. Populations. Am J Prev Med. 2020;58(4):487-95.

149. Wateska AR, Nowalk MP, Jalal H, Lin CJ, Harrison LH, Schaffner W, et al. Is further research on adult pneumococcal vaccine uptake improvement programs worthwhile? Alpha value of information analysis. Vaccine. 2021;39(27):3608-13.

150. Wateska AR, Patricia Nowalk M, Lin CJ, Harrison LH, Schaffner W, Zimmerman RK, et al. Cost-effectiveness of revised US pneumococcal vaccination recommendations in underserved minority adults < 65-years-old. Vaccine. 2022;40(50):7312-20.

151. Willem L, Blommaert A, Hanquet G, Thiry N, Bilcke J, Theeten H, et al. Economic evaluation of pneumococcal vaccines for adults aged over 50 years in Belgium. Hum Vaccin Immunother. 2018;14(5):1218-29.

152. Wolff E, Storsaeter J, Ortqvist A, Naucler P, Larsson S, Lepp T, et al. Cost-effectiveness of pneumococcal vaccination for elderly in Sweden. Vaccine. 2020;38(32):4988-95.

153. Wong CKH, Liao Q, Guo VYW, Xin Y, Lam CLK. Cost-effectiveness analysis of vaccinations and decision makings on vaccination programmes in Hong Kong: A systematic review. Vaccine. 2017;35(24):3153-61.

154. Woolfrey KG. Pneumonia in adults: the practical emergency department perspective. Emerg Med Clin North Am. 2012;30(2):249-70, vii.

155. Wu DB, Rinaldi F, Huang YC, Chang JA, Chang CJ. Economic evaluation of universal 7-valent pneumococcal conjugate vaccination in Taiwan: a cost-effectiveness analysis. J Formos Med Assoc. 2013;112(3):151-60.

156. Xing N, Cheung WY, Jiang M, You JHS. Standing orders program of pneumococcal vaccination for hospitalized elderly patients in Hong Kong: A cost-effectiveness analysis. Am J Infect Control. 2019;47(11):1302-8.

157. Zeevat F, van der Schans J, Boersma WG, Boersma C, Postma MJ. Cost-effectiveness analysis on elderly pneumococcal vaccination in the Netherlands: Challenging the Dutch Health Council's advice. Vaccine. 2019;37(43):6282-4.

158. Zhao D, Gai Tobe R, Cui M, He J, Wu B. Cost-effectiveness of a 23-valent pneumococcal polysaccharide vaccine immunization programme for the elderly in Shanghai, China. Vaccine. 2016;34(50):6158-65.

159. Zhou H, He J, Wu B, Che D. Cost-effectiveness analysis of routine 13-valent pneumococcal conjugate vaccinations in Chinese infants. Hum Vaccin Immunother. 2018;14(6):1444-52.

160. Zou X, He J, Zheng J, Liang M, Gao J, Huang J, et al. Evaluation of effectiveness, safety and cost-benefit of the 23- valent pneumococcal capsular polysaccharide vaccine for HIV-Infected patients. Vaccine. 2022;40(1):37-42.
